# Human pathogens associated with the blacklegged tick *Ixodes scapularis*: a systematic review

**DOI:** 10.1186/s13071-016-1529-y

**Published:** 2016-05-05

**Authors:** Mark P. Nelder, Curtis B. Russell, Nina Jain Sheehan, Beate Sander, Stephen Moore, Ye Li, Steven Johnson, Samir N. Patel, Doug Sider

**Affiliations:** Enteric, Zoonotic and Vector-borne Diseases; Communicable Diseases, Emergency Preparedness and Response; Public Health Ontario, Toronto, Ontario Canada; Institute of Health Policy, Management and Evaluation, University of Toronto, Toronto, ON Canada; Institute for Clinical Evaluative Sciences, Toronto, ON Canada; Analytic Services, Knowledge Services, Public Health Ontario, Toronto, ON Canada; Public Health Ontario Laboratories, Public Health Ontario, Toronto, ON Canada; Department of Laboratory Medicine and Pathobiology, University of Toronto, Toronto, ON Canada; Department of Clinical Epidemiology and Biostatistics, McMaster University, Hamilton, ON Canada

**Keywords:** Bacteria, Blacklegged ticks, Infectious disease, *Ixodes*, Parasites, Pathogens, Public health, Symbionts, Vector-borne, Viruses, Zoonoses

## Abstract

**Background:**

The blacklegged tick *Ixodes scapularis* transmits *Borrelia burgdorferi* (*sensu stricto*) in eastern North America; however, the agent of Lyme disease is not the sole pathogen harbored by the blacklegged tick. The blacklegged tick is expanding its range into areas of southern Canada such as Ontario, an area where exposure to blacklegged tick bites and tick-borne pathogens is increasing. We performed a systematic review to evaluate the public health risks posed by expanding blacklegged tick populations and their associated pathogens.

**Methods:**

We followed PRISMA (Preferred Reporting Items for Systematic Reviews and Meta-Analyses) guidelines for conducting our systematic review. We searched Ovid MEDLINE, Embase, BIOSIS, Scopus and Environment Complete databases for studies published from 2000 through 2015, using subject headings and keywords that included “*Ixodes scapularis*”, “*Rickettsia*”, “*Borrelia*”, “*Anaplasma*”, “*Babesia*” and “pathogen.” Two reviewers screened titles and abstracts against eligibility criteria (i.e. studies that included field-collected blacklegged ticks and studies that did not focus solely on *B. burgdorferi*) and performed quality assessments on eligible studies.

**Results:**

Seventy-eight studies were included in the final review, 72 were from the US and eight were from Canada (two studies included blacklegged ticks from both countries). Sixty-four (82 %) studies met ≥ 75 % of the quality assessment criteria. Blacklegged ticks harbored 91 distinct taxa, 16 of these are tick-transmitted human pathogens, including species of *Anaplasma*, *Babesia*, *Bartonella*, *Borrelia*, *Ehrlichia*, *Rickettsia*, *Theileria* and *Flavivirus*. Organism richness was highest in the Northeast (Connecticut, New York) and Upper Midwest US (Wisconsin); however, organism richness was dependent on sampling effort. The primary tick-borne pathogens of public health concern in Ontario, due to the geographic proximity or historical detection in Ontario, are *Anaplasma phagocytophilum*, *Babesia microti*, *B. burgdorferi*, *Borrelia miyamotoi*, deer tick virus and *Ehrlichia muris*-like sp. Aside from *B. burgdorferi* and to a much lesser concern *A. phagocytophilum*, these pathogens are not immediate concerns to public health in Ontario; rather they represent future threats as the distribution of vectors and pathogens continue to proliferate.

**Conclusions:**

Our review is the first systematic assessment of the literature on the human pathogens associated with the blacklegged tick. As Lyme disease awareness continues to increase, it is an opportune time to document the full spectrum of human pathogens transmittable by blacklegged ticks.

**Electronic supplementary material:**

The online version of this article (doi:10.1186/s13071-016-1529-y) contains supplementary material, which is available to authorized users.

## Background

The blacklegged tick *Ixodes scapularis* is the vector of *Borrelia burgdorferi* (*sensu stricto*) (agent of Lyme disease) in eastern North America. Blacklegged ticks are three-host, non-nidiculous ticks with larvae and nymphs that feed on small rodents (e.g. white-footed mouse) and passerine birds, and adults that feed on large mammals (white-tailed deer, humans). The blacklegged tick’s range has been expanding northward from its precinctive habitats in the Northeast and Upper Midwest US over the last several decades [1–3]. In Ontario, this range expansion has not been uniform; spreading primarily into suburban and rural areas with mixed deciduous forests, where vertebrate hosts are abundant and local climate is favourable to blacklegged tick survival [4]. Until the mid-1990s, *B. burgdorferi* and *Babesia microti* (babesiosis) were the only pathogens known to be transmitted by blacklegged ticks [5–7]. Blacklegged ticks were soon implicated as vectors of *Anaplasma phagocytophilum* (anaplasmosis), and more recently, *Borrelia miyamotoi* (*B. miyamotoi* disease) and deer tick virus (DTV; DTV encephalitis) [8–10]. Uncommon in Ontario’s blacklegged ticks, so far, these pathogens are likely to become more prevalent in the future, as has been the case in recently-invaded jurisdictions such as Maine [11].

In order to assess the public health risks due to blacklegged ticks, an understanding of blacklegged tick-associated organisms is essential, particularly their distribution, prevalence and capacity to cause human disease. We performed a systematic review of the scientific literature to identify organisms (targeting human pathogens) associated with blacklegged ticks in eastern North America and to assess which organisms pose a threat to the health of Ontarians.

## Methods

### Search strategy

We followed PRISMA (Preferred Reporting Items for Systematic Reviews and Meta-Analyses) guidelines for conducting our systematic review [12]. With the assistance of Public Health Ontario’s Library Services, we developed our primary search strategy in Medline and customized it for other databases to account for database-specific vocabulary and functionality. Our search used subject headings and keywords that included “North America”, “*Ixodes scapularis*”, “*Rickettsia*”, “*Bartonella*”, “*Borrelia*”, “*Anaplasma*”, “*Babesia*”, “Powassan”, “pathogen” and “blacklegged tick.” We conducted a systematic review of English-language studies using five electronic databases: Ovid MEDLINE(R) In-Process & Other Non-Indexed Citations and Ovid MEDLINE(R) (Ovid platform: 1 January 1995 to 20 April 2015); Embase (Ovid platform: 1 January 1996 to Week 16, 2015); Scopus (1 January 1999 to 20 April 2015); Environment Complete (EBSCOhost Research Databases: 1 January 1995 to 20 April 2015); and BIOSIS Previews (Ovid platform: 1 January 2002 to Week 20, 2015). All searches are current as of 20 April 2015 (full search strategy for Ovid Medline, Additional file 1).

### Study selection

**Fig. 1:**
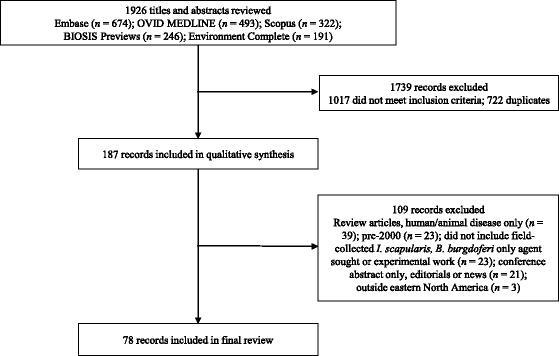
Literature search and study selection

Studies included in the review were required to meet the following eligibility criteria: (i) included field-collected *I. scapularis* from Canada or US; (ii) published on or after 1 January 1995; and (iii) did not test solely for *B. burgdorferi*. We limited studies to those that tested for at least one organism besides *B. burgdorferi* as our emphasis was on the incidence and prevalence of organisms other than *B. burgdorferi*. Studies that focused on experimental models–in the absence of field-collected blacklegged ticks–were excluded (e.g. modeling studies, studies with experimental infections of hosts or ticks), as were studies concentrating on human case reports, patient treatment or blood-donor screening (as these did not specifically link an organism to field-collected blacklegged ticks). We limited the final number of studies to those published on or after 1 January 2000; we did not identify any additional organisms from studies published from 1 January 1995 through 31 December 1999. Two reviewers independently screened titles and abstracts against eligibility criteria and differences were resolved by consensus (Mark P Nelder, Nina Jain Sheehan) (Fig. 1). We excluded one study because it did not identify the state(s) where the ticks were collected [13].

### Data extraction and quality assessment

We populated a data extraction table with the study’s first author, year(s) of study, location of study, tick or host collection methods, organisms tested for (gene targets), key organism detection methods, prevalence of organisms (by tick stage), explicit mention of positive and negative pathogen controls, acknowledgement of sequence submissions to GenBank (where the organism identification was uncertain) (Additional file 2).

To evaluate the quality of eligible studies and to reduce the risk of bias, critical appraisals for each study were completed by two independent reviewers and disagreements resolved by consensus (Curtis B Russell, Mark P Nelder; Additional file 3). We completed critical appraisals of studies using the Public Health Ontario Meta-tool for Quality Appraisal of Public Health Evidence, a composite tool based on multiple underlying instruments (e.g. STROBE, STrengthening the Reporting of OBservational studies in Epidemiology) [14]. We assessed all studies based upon relevancy, reliability, validity and applicability. The independent reviewers did not calculate an overall quality score, in keeping with the agreement in the literature [12].

### Data synthesis and analysis

**Fig. 2:**
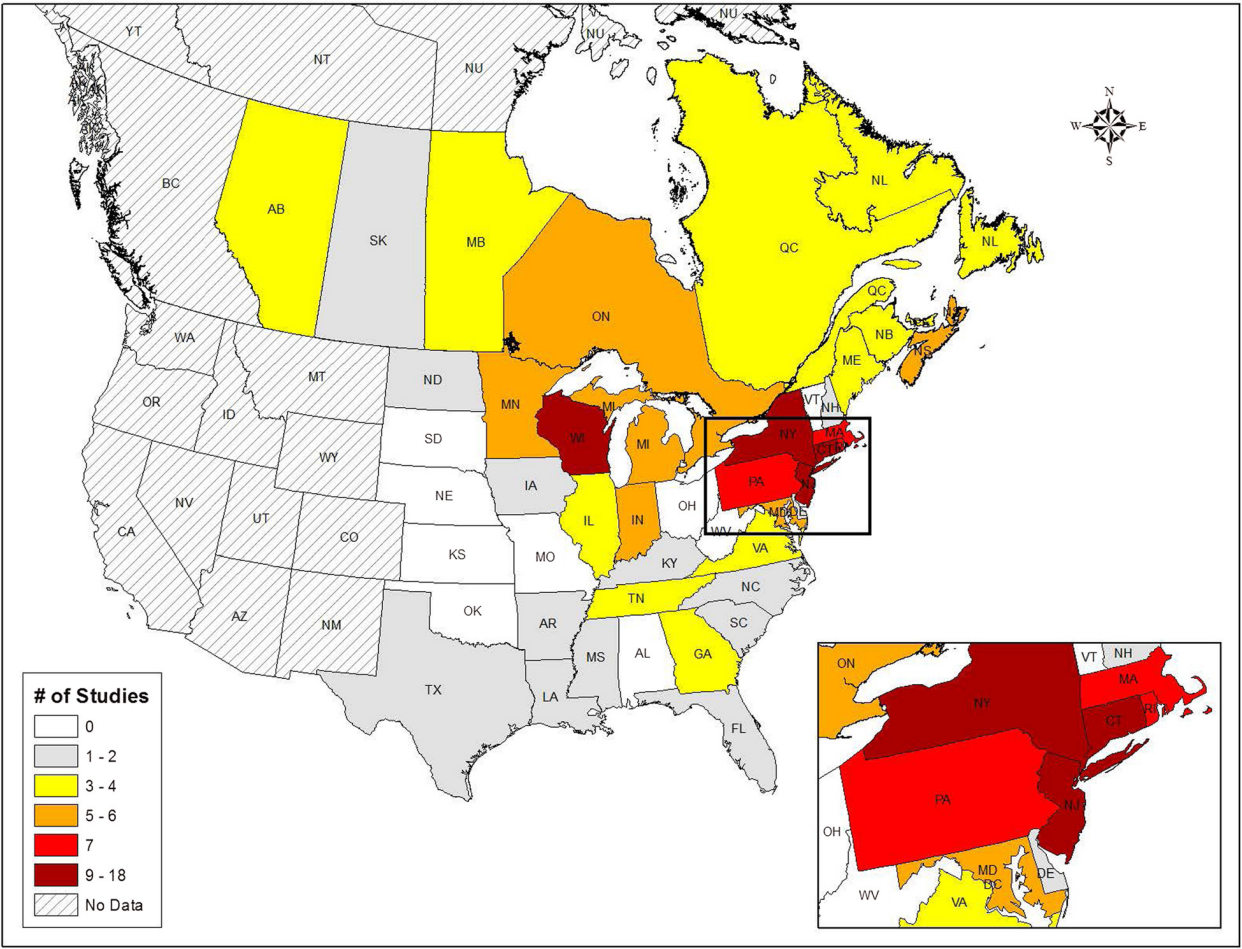
Number of studies performed in each province or state from reviewed studies (2000–2015). No Data, no studies from these jurisdictions were included in the review

**Fig. 3:**
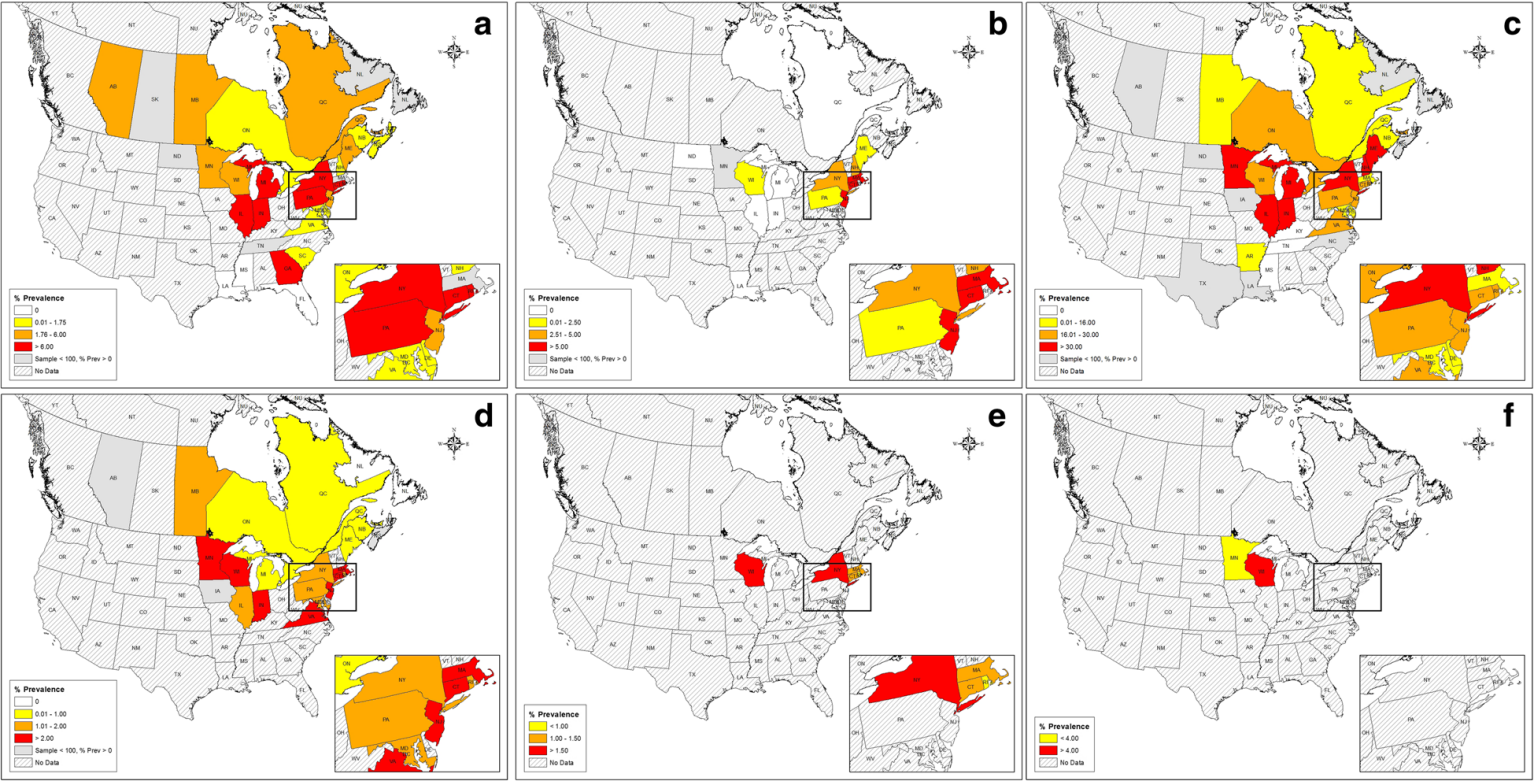
Distribution and crude prevalence for human pathogens transmitted by *Ixodes scapularis* in eastern North America (2000–2015). **a** *Anaplasma phagocytophilum*, **b** *Babesia microti*, **c** *Borrelia burgdorferi*, **d** *Borrelia miyamotoi*, **e** DTV/POWV, **f** *Ehrlichia muris-like*

We structured study outcomes by organism detected, jurisdiction (province or state) where detected and crude prevalence (Table 1; Figs. 2 and 3). Pathogen prevalence is reported as a crude prevalence, indicating the prevalence reported includes pooled tick stages (larvae, nymphs and adults), along with pooled engorgement status, collection methods and pathogen detection methods. To determine what tick-borne diseases are reportable to public health officials, we reviewed reportable disease lists for *Anaplasma*, *Babesia*, *Bartonella*, *Borrelia*, DTV, *Ehrlichia* and *Rickettsia* in Canada (Manitoba, Ontario and Quebec) and the US (Illinois, Indiana, Michigan, Minnesota, New York, Ohio, Pennsylvania and Wisconsin) (Table 2). We created maps presenting crude prevalence (total positive ticks/total ticks tested in a province or state) for the pathogens of concern (Figs. 2 and 3) using the Environmental Systems Research Institute (ESRI) ArcMap Geographic Information System software (v10.3.1), manually choosing data classes, cut-off values and map colours for each of the generated maps. We calculated Pearson product-moment correlation coefficients (significance determined by linear regressions) to test the linear association between the number of organisms detected and the number of (i) studies conducted in a jurisdiction, (ii) ticks tested in a jurisdiction and (iii) organisms sought in a jurisdiction.

**Table 1:** Summary of tick-borne, reportable pathogens and diseases for select provinces and states^a^

Country (province or state)	Arboviruses	*Anaplasma*	*Babesia*	*Bartonella*	*Borrelia*	*Ehrlichia*	*Rickettsia*
Canada							
Ontario [120]	Encephalitis (primary viral)	NR	NR	NR	Lyme disease (*B. burgdorferi*)	NR	NR
Manitoba [121]	NR	Anaplasmosis (*A. phagocytophilum*)	Babesiosis (*Babesia* spp.)	NR	Lyme disease (*B. burgdorferi*)	NR	NR
Quebec [122]	Encephalitis (arthropod-borne)	NR	NR	NR	Lyme disease (*B. burgdorferi*)	Ehrlichiosis (*E. chaffeensis*)	NR
US							
Illinois [123]	Arboviruses	Anaplasmosis	Babesiosis	NR	Lyme disease	Ehrlichiosis	RMSF
Indiana [124]	Encephalitis (POWV)	*A. phagocytophilum*	Babesiosis (*Babesia* spp.)	NR	Lyme disease (*B. burgdorferi*)	Ehrlichiosis (*E. chaffeensis*)	RMSF, other spotted fevers (*Rickettsia* spp.)
Michigan [125]	Arboviral encephalitides (POWV)	Anaplasmosis (*A. phagocytophilum*)	Babesiosis (*Ba. microti*)	Cat-scratch fever (*Bartonella* spp.)	Lyme disease (*B. burgdorferi*)	*Ehrlichia* spp.	Spotted fevers (*Rickettsia* spp.)
Minnesota [126]	Arboviral disease (POWV)	Anaplasmosis (*A. phagocytophilum*)	Babesiosis (*Babesia* spp.)	Cat-scratch disease (*Bartonella* spp.)	Lyme disease (*B. burgdorferi*)	Ehrlichiosis (*E. chaffeensis, E. muris-*like sp. and *E. ewingii*)	RMSF, other spotted fevers (*R. rickettsii*), *Rickettsia* spp.
New York [127]	Arboviral infection (POWV)	Anaplasmosis (*A. phagocytophilum*)	Babesiosis (*Babesia* spp.)	NR	Lyme disease (*B. burgdorferi*)	Ehrlichiosis (*Ehrlichia* spp.)	RMSF, other spotted fevers (*R. akari*, *R. rickettsii*)
Ohio [128]	Arbovirus (POWV)	Anaplasmosis	Babesiosis	NR	Lyme disease	Ehrlichiosis	RMSF, other spotted fevers (*Rickettsia* spp.)
Pennsylvania [129]	Arboviruses	Anaplasmosis	NR	NR	Lyme disease	Ehrlichiosis	RMSF, rickettsialpox (*Rickettsia* spp.)
Wisconsin [130]	Arboviral infection (encephalitis, meningitis)	Anaplasmosis	Babesiosis	NR	Lyme disease	Ehrlichiosis	RMSF

^a^NR, not reportable; RMSF, Rocky Mountain spotted fever; POWV, Powassan encephalitis virus

**Table 2:** Distribution of *Ixodes scapularis* organisms in eastern North America, from 78 studies reviewed (2000–2015)

Organism	Jurisdiction where organism detected (Canadian provinces and US states)^a^
Tick-borne organisms	
Alphaproteobacteria	
Anaplasmataceae	
* Anaplasma phagocytophilum* ^*b*,c^	Canada: AB, MB, NB, NL, NS, ON, PE, QC, SK; US: CT, DE, GA, IL, IN, MA, MD, ME, MI, MN, NC, ND, NH, NJ, NY, PA, RI, SC, TN, VA, WI
* Ehrlichia chaffeensis* ^*b*^	US: NY
* Ehrlichia ewingii* ^*b*^	US: TN
* Ehrlichia* Panola Mountain sp.^*b*^	US: TN
* Ehrlichia muris*-like sp. (or *E.* sp. nr. *muris*)^*b*^	US: MN, WI
Bartonellaceae	
* Bartonella henselae* ^*b*^	US: NJ, NY, PA
Unidentified/uncharacterized * Bartonella* spp.	US: MD, NJ, PA
Rickettsiaceae	
* Candidatus* Rickettsia cooleyi (or cooleyi-like)^d^	US: FL, GA, TN, TX
* Candidatus* Rickettsia IRS4	US: MA
* Candidatus* Rickettsia amblyommii	US: TN
* Rickettsia buchneri* ^d^	US: MN
* Rickettsia massiliae*/*Rickettsia* sp. Bar 29	US: NC
* Rickettsia parkeri* ^*b*^	US: LA
* Rickettsia peacockii* ^d^	US: TX
* Rickettsia* endosymbiont of * Ixodes scapularis* ^d^	US: CT, IN, LA, MA, ME, NY, PA, TN, WI
* Rickettsia* sp. Is-I	US: GA
* Rickettsia* sp. TR-39	US: FL, GA
Unidentified/uncharacterized * Rickettsia* spp.	US: MD, NC, TN
* Wolbachia* sp.	US: CT
Spirochaetes	
Spirochaetaceae	
* Borrelia andersonii* ^*b*^	US: MI
* Borrelia bissettii* ^*b*^	US: LA
* Borrelia burgdorferi* s.s.^*b*^	Canada: AB, MB, NB, NS, NL, ON, PE, QC; US: AR, CT, DE, GA, IA, IL, IN, LA, MA, MD, ME, MI, MN, NC, ND, NH, NJ, NY, PA, RI, SC, TX, VA, WI
* Borrelia kurtenbachii* ^*b*^	Canada: NS; US: NY
* Borrelia lonestari* ^*b*^	US: AR, MA, NY
* Borrelia miyamotoi* ^*b*^	Canada: AB, MB, NB, NS, ON, PE, QC; US: CT, IA, IL, IN, MA, MD, ME, MI, MN, NJ, NY, PA, RI, VA, WI
Unidentified/uncharacterized * Borrelia* sp.	US: NY
Piroplasmida	
Babesiidae	
* Babesia microti* ^*b*^	US: CT, MA, MD, ME, MN, NH, NJ, NY, PA, WI
* Babesia odocoilei*	US: IN, IL, ME, MI, MS, PA, TN, WI
Theileriidae	
* Theileria cervi*	US: TN
Viruses	
Bunyaviridae	
Blacklegged tick phlebovirus	US: NY
South Bay virus	US: NY
Flaviviridae	
Deer tick virus (POWV lineage II)^*b*^	US: CT, MA, NY, RI, WI
Powassan virus (POWV lineage I)^*b*^	US: NY
POWV (not subtyped)^*b*^	US: NY
Reoviridae	
St. Croix River virus^e^	US: WI
Other	
* Ixodes scapularis* mononegavirales^e^	US: NY
Non-vector-borne, gut/surface orgnaisms	
Alphaproteobacteria	
Bradyrhizobiaceae	
* Afipia broomeae* ^f^	US: NY
* Afipia felis* ^f^	US: CT
Caulobacteraceae	
Uncultured bacterium 1	US: CT
Uncultured bacterium 2	US: CT
Methylobacteriaceae	
* Methylobacterium mesophilicum* ^f^	US: CT
* Methylobacterium* sp. strain G296-5	US: CT
Uncultured bacterium 3	US: CT
Sphingomonadaceae	
* Sphingomonas elodea* ^f^	US: CT
* Sphingomonas melonis* ^f^	US: CT
* Sphingomonas* sp.	US: CT
* Sphingomonas* sp. strain BF2	US: CT
* Sphingomonas* sp. AV069	US: CT
* Sphingopyxis alaskensis*	US: CT
* Sphingobacterium* sp.	US: NY
Betaproteobacteria	
Burkholderiaceae	
* Burkholderia* sp.	US: CT
* Ralstonia mannitolilytica* ^f^	US: NY
Comamonadaceae	
* Delftia acidovorans* ^f^	US: CT
* Delftia acidovorans* WDL34^f^	US: CT
Uncultured bacterium 4	US: CT
Uncultured bacterium 5	US: CT
Gammaproteobacteria	
Enterobacteriaceae	
* Enterobacter asburiae* ^f^	US: NY
* Escherichia coli* ^f^	US: CT
* Photorhabdus* sp.	US: NY
* Raoultella* sp.	US: NY
* Shigella* sp.	US: NY
Uncultured gamma protebacterium	US: NY
* Acinetobacter* sp. strain phenon 4	US: CT
* Acinetobacter* sp. 1	US: CT
* Acinetobacter* sp. 2	US: NY
Pseudomonadaceae	
* Pseudomonas fluorescens* ^f^	US: NY
* Pseudomonas* sp. 1	US: NY
* Pseudomonas* sp. 2	US: NY
* Pseudomonas* sp. 3	US: NY
Symbiont cf. *Pseudomonas*	US: NY
Xanthomonadaceae	
* Stenotrophomonas maltophilia* ^f^	US: CT
Uncultured *Stenotrophomonas* sp.	US: NY
* Stenotrophomonas* sp.	US: NY
Actinobacteria	
Mycobacteriaceae	
* Mycobacterium manitobense* ^f^	US: CT
Nocardiaceae	
* Rhodococcus erythropolis* strain NVI^f^	US: CT
* Rhodococcus* sp.	US: NY
* Williamsia* sp.	US: NY
Propionibacteriaceae	
* Propionibacterium acnes* ^f^	US: CT
Firmicutes	
Bacillaceae	
* Bacillus* sp.	US: CT
Streptococcaceae	
Uncultured *Streptococcus* sp.	US: CT
Unclassified Bacteria	
Uncultured bacterium 6	US: CT
Uncultured bacterium 7	US: CT
Uncultured bacterium 8	US: CT
Uncultured bacterium 9	US: CT
Uncultured bacterium 10	US: CT
Uncultured bacterium 11	US: CT
Uncultured bacterium 12	US: CT
Uncultured bacterium 13	US: CT
Plastid clone	US: CT
Uncultured bacterium 14	US: NY
Uncultured/unidentified bacterium	US: NY

^a^Canada: AB, Alberta; MB, Manitoba; NB, New Brunswick; NL, Newfoundland; NS, Nova Scotia; ON, Ontario; PE, Prince Edward Island; SK, Saskatchewan; QC, Quebec. US: AR, Arkansas; CT, Connecticut; DE, Delaware; FL, Florida; GA, Georgia; IL, Illinois; IN, Indiana; IA, Iowa; LA, Louisiana; ME, Maine; MD, Maryland; MA, Massachusetts; MI, Michigan; MN, Minnesota; MS, Mississippi; NH, New Hampshire; NJ, New Jersey; NY, New York; NC, North Carolina; ND, North Dakota; PA, Pennsylvania; RI, Rhode Island; SC, South Carolina; TN, Tennessee; TX, Texas; VT, Vermont; VA, Virginia; WI, Wisconsin

^b^Known or putative human pathogen, *B. andersoni* [131]; *B. bissettii* [132]; *B. kurtenbachii* [133]; *B. lonestari* [134]; *R. parkeri* [91]

^c^Both the *Ap-ha* strain (human disease strain) and the *Ap-variant-1* strain were pooled during data extraction, since not all studies distinguished between the two

^d^Known or putative endosymbiont of *Ix. scapularis*

^e^Likely a viral genome integrated into *I. scapularis* genome [135, 136]

^f^Human pathogen, but likely not transmitted by blacklegged ticks, gut organisms

## Results

### Study characteristics

Seventy-eight studies were included in our final review (Additional file 2) [4, 8, 15–90]. Eight studies included blacklegged ticks from Canada and 72 studies included samples from the US (two studies included Canadian and US blacklegged ticks). In Canada, Ontario (*n* = 6) was sampled the most frequently, followed by Nova Scotia (*n* = 5) (Fig. 2). In the US, New York (*n* = 18) was sampled the most frequently, followed by Wisconsin (*n* = 12), New Jersey (*n* = 10), Connecticut (*n* = 9), Massachusetts (*n* = 7), Pennsylvania (*n* = 7) and Rhode Island (*n* = 7) (Fig. 2). Most Canadian studies did not provide details on the sub-provincial regions where tick collection occurred; however, the areas sampled most frequently were southern regions of Ontario and Nova Scotia. In the US, counties that were sampled the most were Dutchess (New York) (*n* = 11), Westchester (New York) (*n* = 9), Monmouth (New Jersey) (*n* = 6) and New London (Connecticut) (*n* = 6). No studies included blacklegged ticks from Alabama, Kansas, Missouri, Ohio, Oklahoma, South Dakota, Vermont or West Virginia. From 2000 through 2007, three studies were published per year (range = 2–4); from 2009 through 2015, 6.8 studies were published per year (range = 1–19).

### Quality assessment

Twenty studies (26 % or 20 out of 78) met 100 % of quality assessment criteria and an additional 44 studies (56 %) met 75 % of criteria (Additional file 3). Thirty-three studies (42 %) collected blacklegged ticks by dragging or flagging; 21 (27 %) collected ticks from mammal or bird hosts; 15 (19 %) used a combination of dragging and animal collections; and nine (12 %) did not report collection methods or employed other techniques. Thirty-three studies (42 %) explicitly reported positive and negative controls; four (5 %) reported negative controls only; 10 (13 %) reported positive controls only; and 31 (40 %) did not report any controls (Additional file 2). Six studies (38 % or six out of 16) included GenBank accession numbers for gene sequenced PCR products where species identification was indeterminate.

### Descriptive analysis

Blacklegged ticks harbored 91 distinct taxa. Sixteen of these organisms are tick-transmitted human pathogens; the remainder is primarily gut or surface microbes of blacklegged ticks and not transmittable to humans or symbionts of blacklegged ticks (Table 1). The highest number of organisms detected in the Canadian-sampled blacklegged ticks were from Nova Scotia (*n* = 4; i.e. at least four separate identifications of the organisms sought in the ticks sampled), followed by three each in Alberta, Manitoba, New Brunswick, Ontario and Prince Edward Island (Table 2). US-sampled blacklegged ticks harbored 91 organisms. The highest number of organisms were detected in Connecticut (*n* = 42), followed by New York (*n* = 36), Wisconsin (*n* = 9), Tennessee (*n* = 9), Massachusetts (*n* = 8) and Pennsylvania (*n* = 8). Studies in different jurisdictions sought varying numbers of pathogens and often used various detection methods. The mean ± SE number of studies per jurisdiction (4.2 ± 0.60; median = 3; *n* = 78) was positively correlated (*r* = 0.77, *n* = 37, *P* < 0.0001) with the mean ± SE number of organisms detected per jurisdiction (5.2 ± 1.09; median = 4; *n* = 78). Similarly, the number of blacklegged ticks tested in a jurisdiction (mean = 2,797 ± 1,022.6; median = 696) was positively correlated with the number of organisms detected per jurisdiction (*r* = 0.72, *n* = 37, *P* < 0.0001). In the studies reviewed, researchers performed 149 unique sampling events, where a sampling event is the testing of blacklegged ticks for a specific organism in a specific jurisdiction. The number of organisms sought per sampling event (mean = 2.8 ± 0.29; median = 2; *n* = 149) was positively correlated (*r* = 0.95, *n* = 149, *P* < 0.0001) with the number of organisms detected per sampling event (mean = 2.3 ± 0.28; median = 2; *n* = 149). We performed multiple tests on the same dataset, however, since these *P*-values are very small, adjusting for multiple testing would not alter the statistical significance of the results.

Thirty-two distinct organisms transmitted by ticks were reported in the studies reviewed (Table 1). Five species of Anaplasmataceae were detected; *A. phagocytophilum* was most prevalent in Rhode Island (21.3 %; *n* = 684), Georgia (17.3 %; *n* = 910), Connecticut (13.0 %; *n* = 454), Indiana (10.8; *n* = 712), New York (8.9 %; *n* = 25,098), Michigan (7.9 %; *n* = 444), Pennsylvania (6.5 %; *n* = 1,559) and Illinois (6.5 %; *n* = 278) (Fig. 3; Table 1). *Ehrlichia muris*-like sp. was restricted to Wisconsin (5.4 %; *n* = 4,066) and Minnesota (3.0 %; *n* = 534) (Fig. 3). Blacklegged ticks harbored one Bartonellaceae species, *Bartonella henselae*, which was only found in ticks from New Jersey (100 %; *n* = 1), New York (2.3 %; *n* = 88) and Pennsylvania (3.1 %; *n* = 544). Blacklegged ticks contained 12 *Rickettsia* taxa; *Rickettsia* endosymbiont of *Ixodes scapularis*, a blacklegged tick obligate endosymbiont (non-pathogenic), was the most prevalent taxa, particularly in Pennsylvania (64.9 %; *n* = 94), Indiana (63.0 %; *n* = 100), Tennessee (51.1 %; *n* = 47) and Maine (46.0 %; *n* = 100). Seven out of 12 *Rickettsia* species were restricted geographically to the Southern US and the known human pathogen *Rickettsia parkeri* was limited to Louisiana (16.6 %; *n* = 18). Ticks sampled across all 78 studies contained six species of *Borrelia. Borrelia burgdorferi* was most prevalent in New Hampshire (52.3 %; *n* = 509), Maine (49.1 %; *n* = 10,004), Minnesota (47.2 %; *n* = 803), Indiana (45.3 % *n* = 506), Michigan (39.5 %; *n* = 696), Illinois (33.9 %; *n* = 460) and New York (31.6 %; *n* = 21,363). *Borrelia miyamotoi* was most prevalent in Connecticut (4.7 %; *n* = 1,226), Indiana (4.1 %; *n* = 487), Virginia (3.5 %; *n* = 173), Minnesota (2.9 %; *n* =700), New Jersey (2.7 %; *n* = 765), Massachusetts (2.5 %; *n* = 159) and Wisconsin (2.4 %; *n* = 3,151) (Fig. 3). Blacklegged ticks harbored three Apicomplexan parasites; *Ba. microti* was most prevalent in Connecticut (6.7 %; *n* = 1,198), New Jersey (6.5 %; *n* = 1.195) and Massachusetts (5.3 %; *n* = 851) (Fig. 3). Several viruses were detected in blacklegged ticks; however, only DTV and Powassan virus (POWV) are of concern as human pathogens. Deer tick virus/POWV was most common in New York (3.4 %; *n* = 91) (Fig. 3). Pathogens sought for but not detected in any samples included *Coxiella burnetii* (New York, *n* = 88), *Francisella tularensis* (New York, *n* = 374; Florida, *n* = 52; Georgia, *n* = 13) and *Rickettsia rickettsii* (New York, *n* = 88) (Additional file 2).

Two of the studies reported 55 additional bacteria taxa, common genera were *Acinetobacter* (Enterobacteriaceae), *Afipia* (Alphaproteobacteria), *Pseudomonas* (Pseudomonadaceae), *Sphingomonas* (Sphingomonadaceae) and *Stenotrophomonas* (Xanthomonadaceae) [19, 64]. While most of the bacteria identified are commonly found in the environment (i.e., soil, water), some are associated with mammals, such as *E. coli*, *Shigella* sp. and *Streptococcus* sp. These additional bacteria are likely a part of the microbial community (as commensal organisms or environmental contaminants) of the blacklegged tick’s gut or surface and not necessarily transmitted or maintained by blacklegged ticks.

The mandatory reporting requirements of pathogens varied among the jurisdictions reviewed. Deer tick virus is typically reportable as an arboviral infection in most of the jurisdictions reviewed except for Manitoba (Table 2). In Canada, anaplasmosis is reportable only in Manitoba, yet reportable in all US states reviewed. In Canada, babesiosis is reportable only in Manitoba, but in all US states reviewed except Pennsylvania. Bartonellosis was not reportable in any Canadian jurisdiction and reportable only in Michigan and Minnesota in the US. Lyme disease (as *B. burgdorferi*) is reportable in all jurisdictions reviewed; however, infection by other *Borrelia* species is not. In Canada, only Quebec requires reporting of ehrlichiosis, but ehrlichiosis is reportable in all US states reviewed. In Canada, rickettsial infections are not reportable, but are reportable in all US states reviewed.

## Discussion

Our systematic review of 78 North American studies published since 2000 documented that blacklegged ticks are associated with 91 distinct organisms. Sixteen of these organisms are tick-transmitted human pathogens. The remainder of the organisms are non-pathogenic gut or surface biota (as commensal organisms or environmental contaminants), intracellular symbionts of ticks or have unknown pathologies in vertebrates. Blacklegged ticks are the principal vectors of six of these human pathogens: *A. phagocytophilum*, *Ba. microti*, *B. burgdorferi*, *B. miyamotoi*, DTV and *Ehrlichia muris*-like sp. Due to studies documenting their proximity to, or detection in, Ontario, the six pathogens represent the primary risks in the province, with two presenting more immediate threats than others due to their recent detection in parts of Ontario (i.e. *A. phagocytophilum*, *B. burgdorferi*). Similar to our review, 20 human pathogens were reported from Europe’s sister taxa to the blacklegged tick, *Ixodes ricinus* [91, 92]. Given the blacklegged tick’s liberal feeding behavior and propensity to bite humans, continued identification of blacklegged tick-pathogen relationships and assessing their public health implications is justified.

Blacklegged ticks transmit two of the Anaplasmataceae bacteria reported in the review (i.e. *A. phagocytophilum* and *Ehrlichia muris*-like sp.). The number of human granulocytic anaplasmosis cases has increased in the US from less than 200 cases in 1997 to over 2,700 cases in 2013, with most cases reported from Massachusetts, Minnesota, New York and Wisconsin [93, 94]. Patients infected with *A. phagocytophilum* display (in order of decreasing incidence) malaise, fever, myalgia, headache, arthralgia and nausea [95]. *Anaplasma phagocytophilum* is relatively rare (< 0.5 %) in Ontario blacklegged ticks and we are not aware of any anaplasmosis cases diagnosed from the province [4]. *Ehrlichia muris*-like sp. is restricted to Wisconsin and Minnesota; however, this pathogen has not been widely tested for elsewhere. Little is known about this pathogen; nevertheless, it has been identified in 73 patients from Indiana, Michigan, Minnesota, North Dakota and Wisconsin (all cases reported tick-bite exposures from Minnesota or Wisconsin) displaying fever, malaise, headache and myalgia [96]. Considering *A. phagocytophilum* is present in Ontario and the distribution of *Ehrlichia muris*-like sp. is not well-characterized, awareness of these pathogens by public health officials is warranted.

We expected a relatively high number of rickettsial agents to be reported from blacklegged ticks, given the extensive association of *Rickettsia* with hematophagous invertebrates such as fleas (Siphonaptera) and other ticks (Ixodidae, Argasidae) [91]. Human disease has not been associated with the majority of rickettsial organisms identified in this review; however, careful clinical and epidemiological studies can lead to some of these being identified as human pathogens (e.g. *R. parkeri*) [97]. Currently, there is no evidence demonstrating blacklegged ticks as effective vectors of *Rickettsia*; however, further investigation is justified where unexplained cases of spotted fever occur with tick-bite histories.

The reviewed studies reported that blacklegged ticks harbor six species of *Borrelia*, two of which are primarily reported from blacklegged ticks (i.e. *B. burgdorferi* and *B. miyamotoi*). *Borrelia burgdorferi* was widely distributed throughout the range of the blacklegged tick (at least in the ticks examined in these studies), including Ontario (prevalence = 16 % of blacklegged ticks), with the highest prevalence (> 30 %) in the Northeast and Midwest US. Studies focusing solely on *B. burgdorferi* were not included in review; therefore, caution must be used when interpreting the prevalence of *B. burgdorferi*. Patients with acute *B. burgdorferi* infection usually display erythema migrans (bulls-eye rash), fever, myalgia, headache, arthralgia, neck stiffness and arthritis [98]. In Ontario, the Lyme disease incidence rate has increased from 0.2 (2002) to 1.6 cases per 100,000 population (2014) [99]. *Borrelia miyamotoi* is a newly recognized human pathogen, which based on the reviewed studies has a widespread distribution in North America (Ontario prevalence < 0.5 %). Recently, 53 patients with Lyme disease-like symptoms from Connecticut, Massachusetts, New Jersey, New York and Rhode Island showed serological evidence for *B. miyamotoi* infection [100, 101]. Patients with *B. miyamotoi* disease display fever, fatigue, headache, myalgia, chills and nausea [10, 102]. To our knowledge, *B. miyamotoi* disease has not been diagnosed in Ontario. Since our literature search was performed, a new blacklegged tick-transmitted *Borrelia* has been associated with human disease in Wisconsin, *Candidatus* Borrelia mayonii [103, 104]. *Borrelia burgdorferi* has rightfully been the *Borrelia* of concern to public health; however, other *Borrelia* species are beginning to emerge as additional threats to public health.

While the focus of this systematic review was organisms of public health significance, it is important to note the superficially benign microbes and symbionts associated with blacklegged ticks. While outside the scope of this review, the gut microorganisms of blacklegged ticks can moderate the colonization of human pathogens in blacklegged ticks, such as with *B. burgdorferi* [105, 106]. Several of the gut or surface bacteria (e.g. *Stenotrophomonas maltophilia* and *Rhodococcus erythropolis*) associated with opportunistic infections in humans, especially in immunocompromised individuals [107–109]. Our understanding of the role that symbionts play in the biology of blacklegged ticks and the blacklegged tick’s ability to transmit pathogens is poorly understood and represents an opportunity for future research. Symbionts, such as *Rickettsia* endosymbiont of *Ixodes scapularis* and *Wolbachia* sp. (closely related to *W. pipientis* supergroup A) were reported from the studies reviewed. *Wolbachia* symbionts are involved in the manipulation of arthropod reproduction (e.g. cytoplasmic incompatibility and parthenogenesis) in other arthropods such as *Drosophila* and parasitoid wasps [110]. Understanding how the entire tick microbiome regulates pathogen acquisition and transmission is a burgeoning field, undoubtedly providing insights into potential blacklegged tick management options. In addition, a better understanding of *Ix. scapularis* symbionts could lead to novel management tools.

In the reviewed studies, *Ba. microti* was most common in blacklegged ticks collected from the Coastal Atlantic states of the Northeast US. In the US, the number of babesiosis cases has increased from approximately 1,100 (2011) to 1,800 (2013), with most cases reported from Connecticut, Massachusetts, New Jersey and New York [94, 111]. Patients with babesiosis display fever, fatigue, headache, chills and arthralgia [112]. While *Ba. microti* could eventually occupy the same distribution of *B. burgdorferi*, it will do so at a slower rate due to several factors. For example, blacklegged tick transmission of *Ba. microti* to reservoir hosts is not as efficient when compared to *B. burgdorferi* [113, 114]. In addition, the survival of *Ba. microti* is low in overwintering blacklegged tick nymphs when compared to *B. burgdorferi* [115]. *Babesia microti* has not been detected in Ontario blacklegged ticks (in the published literature) or identified as the cause of locally acquired disease, to our knowledge.

Deer tick virus (POWV lineage II) was the most common viral agent detected in the studies reviewed, a viral species serologically indistinguishable from POWV (POWV lineage I) but with unique nucleotide and amino acid sequences. In 2009, DTV was the cause of a fatal case of encephalitis from New York, the first report implicating DTV as an agent of human disease [116]. Soon after this initial case report, additional cases were reported in New York that displayed fever, malaise, confusion, seizure, headache, rash and vomiting [117]. There is no evidence for the presence of DTV in the Ontario’s blacklegged ticks; however, field research is underway to determine its presence (Curtis B Russell, unpublished data).

Describing pathogens associated with blacklegged ticks is only the first step towards appreciating the role of this tick as North America’s most important vector of public health significance. The majority of pathogens and intracellular symbionts reported here have poorly understood natural histories and, for the most part, we know little of their maintenance in nature, their potential or preferred vertebrate reservoirs and, in some cases, their ability to cause disease in vertebrates. While symbionts and non-human pathogens are relatively benign, it is important to understand their ecology as well, because they likely play roles in determining the distribution and prevalence of the human pathogens discussed through competitive exclusion [118]. In Ontario blacklegged ticks, only a handful of pathogens have been sought after and there is an opportunity to better understand their ecology (e.g. *Babesia*, *Ehrlichia*, *Rickettsia*, DTV). Understanding the complex epidemiology of the human pathogens transmittable by blacklegged ticks will require longitudinal, ecological (host and vector competence) and epidemiological studies in endemic and emerging areas.

While comprehensive, our systematic review does have several limitations. We did not perform a search of the grey literature (publications on testing results from public health or government institutions); therefore, our results might be biased towards primary research with positive results due to publication bias. In Canada, blacklegged ticks collected by passive surveillance were likely used in multiple studies for pathogen detection; however, an examination of the studies shows that overlap (same ticks used in separate studies) involved only two studies each from 2007 through 2012 and was limited to the detection of *A. phagocytophilum* and *B. burgdorferi*. Another limitation is that studies were undertaken where pathogen prevalence or disease burden are high, meaning prevalence does not represent a uniform value across a province or state. The heterogeneity among studies in terms of collection methods, blacklegged tick stages assayed, engorgement levels of ticks, molecular methods used or gene targets undoubtedly influenced the crude prevalences we reported. For example, we expect that ticks collected from reservoir hosts (pathogen acquisition can also vary by host type and tick stage) would have a higher pathogen load, compared to host-seeking ticks collected by dragging. The number of organisms detected in a jurisdiction is dependent upon sampling effort and testing methods; therefore, little-studied areas may indeed hold more blacklegged tick- organisms. The presence of pathogens in neighboring jurisdictions or in Ontario itself provides the basis for assessing the risks of blacklegged tick-transmitted infections in Ontario, but this method does not allow for an accurate estimate of when these pathogens will be a threat to Ontario. As far as we know, blacklegged ticks transmit and maintain all the underscored pathogens within a rodent-mammal-tick cycle in temperate, mixed deciduous forests. Given the pathogens share a common transmission cycle, these pathogens should eventually occur throughout the blacklegged tick’s range. The review identified that most research has occurred in relatively small geographic areas, representing an opportunity to determine pathogen incidence and prevalence outside of highly sampled regions. As research continues to detect pathogens in blacklegged ticks and reservoir hosts, we expect the number of pathogens to increase. In addition, comprehensive studies, seeking all possible pathogens using standardized methods, are needed for better comparison of pathogen prevalence across northeastern North America. Our review is the first systematic assessment of literature, identifying and bringing together the scattered knowledge of human pathogens associated with the blacklegged tick.

## Conclusions

Viewed as a mere pest until the mid-1970s, the blacklegged tick was not known to transmit pathogens to humans or other animals [119]. In the following 40 years, the blacklegged tick has turned into the most important vector in North America. Our review has identified several human pathogens besides *B. burgdorferi* that are transmittable by blacklegged ticks, yet we have not demonstrated disease in Ontario at this time for *A. phagocytophilum*, *Ba. microti*, *B. miyamotoi*, DTV or *Ehrlichia muris*-like sp. While most of these pathogens do not represent immediate public health threats in Ontario, there is an opportunity to ensure mitigation efforts are in place prior to their arrival. The growing public and physician awareness concerning Lyme disease provides a catalyst upon which to synergize awareness of other tick-borne diseases. In addition, surveillance of pathogens in field-collected blacklegged ticks will establish baseline data and inform local risk assessments. Public health in Ontario needs to remain vigilant because Ontario (i) has a relatively higher population of at-risk people in Canada, (ii) has blacklegged tick populations that continue to expand and (iii) is positioned next to jurisdictions where these pathogens occur.

## Additional files

Additional file 1: Search strategy example using Ovid MEDLINE(R) In-Process & Other Non-Indexed Citations and Ovid MEDLINE(R) 1 January 1995 to 20 April 2015. (DOCX 26 kb) Additional file 2: Summary of organisms detected in *Ixodes scapularis* collected throughout eastern North America, from 78 studies reviewed (2000–2015). (DOCX 117 kb) Additional file 3: Critical appraisal of 78 studies in review. (DOCX 61 kb)

## Abbreviations

ESRI: Environmental Systems Research Institute
DTV: deer tick virus
PCR: polymerase chain reaction
POWV: Powassan encephalitis virus
PRISMA: Preferred Reporting Items for Systematic Reviews
NR: not reportable
RMSF: Rocky Mountain spotted fever
STROBE: Strengthening the Reporting of OBservational Studies in Epidemiology
