# Supplementary material for: Human pathogens associated with the blacklegged tick Ixodes scapularis: a systematic review
Source: Parasit Vectors. 2016 May 5;9:265. doi: 10.1186/s13071-016-1529-y (PMC4857413; doi:10.1186/s13071-016-1529-y)
Supplement: Additional file 1: — Search strategy example using Ovid MEDLINE(R) In-Process & Other Non-Indexed Citations and Ovid MEDLINE(R) 1 January 1995 to 20 April 2015. (DOCX 26 kb) [file 13071_2016_1529_MOESM1_ESM.docx]

**Additional file 1.** Search strategy example using Ovid MEDLINE(R) In-Process & Other Non-Indexed Citations and Ovid MEDLINE(R) 1 January 1995 to 20 April 2015

| # | Searches | Results |
| --- | --- | --- |
| 1 | north america/ or canada/ or manitoba/ or new brunswick/ or nova scotia/ or ontario/ or quebec/ or united states/ or appalachian region/ or maryland/ or new york/ or ohio/ or pennsylvania/ or tennessee/ or virginia/ or great lakes region/ or illinois/ or indiana/ or michigan/ or minnesota/ or wisconsin/ or mid-atlantic region/ or delaware/ or new jersey/ or new england/ or connecticut/ or maine/ or massachusetts/ or new hampshire/ or rhode island/ or vermont/ | 1006578 |
| 2 | (canada or ontario or quebec or new brunswick or nova scotia or manitoba or united states or northeast or upper Midwest or hudson valley or new york or new jersey or maine or connecticut or vermont or new england or eastern seaboard or rhode island or massachusetts or new hampshire or michigan or ohio or minnesota or illinois or indiana or pennsylvania or tennessee).tw,kf,kw. | 361401 |
| 3 | (beaudry or pembina or st malo or saint malo or roseau river or winnipeg or arbaka or assiniboine or beaudry or pointe-pelee or pelee or rondeau or turkey point or long point or wainfleet bog or prince edward point or thousand islands or saint lawrence valley or st lawrence valley or ottawa or pinery park or "lake of the woods" or monteregie or estrie or millidgeville or saint john or north head or grand manan island or halifax or lunenburg or shelburne or yarmouth or pictou).tw,kf,kw. | 4776 |
| 4 | 1 or 2 or 3 | 1196726 |
| 5 | ticks/ or ixodidae/ or tick infestations/ or tick bites/ or ixodes/ or (ixodes scapularis or i scapularis or black legged tick? or blacklegged tick? or ixod$ tick? or ixode? or bear tick? or deer tick?).tw,kf,kw. | 17213 |
| 6 | tick-borne diseases/ or ehrlichiosis/ or Ehrlichia/ or Tularemia/ or Francisella tularensis/ or Babesiosis/ or Encephalitis, Tick-Borne/ or Anaplasmosis/ or Borrelia infections/ or Babesia microti/ or Encephalitis Viruses, Tick-Borne/ or Anaplasma phagocytophilum/ or Borrelia/ or Rickettsia/ or Rickettsia infections/ | 21058 |
| 7 | (((tick bo?rne or tickbo?rne or powassan or POW) adj1 (pathogen? or zoonoses or disease? or encephalitis or virus$)) or deer tick virus$ or lyme disease-like or (borrelia adj (bissettii or carolinensis or kurtenbachii or microti or miyamotoi)) or (b adj (odocoilei or bissettii or carolinensis or kurtenbachii or microti or miyamotoi)) or POWV or a phagocytophilum or human granulocytic anaplasmosis or HGA or e phagocytophilum or ehrlichia phagocytophilum or human monocytic ehrlichiosis or HME or ehrlichia chaffeensis or ehrlichia ewingii or francisella tularensis or e chaffeensis or e ewingii or f tularensis or tularemia or babesia microti or babesia odocoilei or rickettsia or DTV or TBEV).tw,kf,kw. | 17075 |
| 8 | 6 or 7 | 27500 |
| 9 | 4 and 5 and 8 | 704 |
| 10 | limit 9 to english language | 678 |
| 11 | limit 10 to last 20 years | 500 |
