# Supplementary material for: Human pathogens associated with the blacklegged tick Ixodes scapularis: a systematic review
Source: Parasit Vectors. 2016 May 5;9:265. doi: 10.1186/s13071-016-1529-y (PMC4857413; doi:10.1186/s13071-016-1529-y)
Supplement: Additional file 2: — Summary of organisms detected in Ixodes scapularis collected throughout eastern North America, from 78 studies reviewed (2000–2015). (DOCX 117 kb) [file 13071_2016_1529_MOESM2_ESM.docx]

**Additional file 2.** Summary of organisms detected in *Ixodes scapularis* collected throughout eastern North America, from 78 studies reviewed

| Study; first author, year published; ref, year(s) of study* | Organism | Stage: prevalence (x/n)** | Pathogen detection  methods†; controls; GenBank submission^‡^ | State or Province (sub-jurisdictions) sampled^‡‡^ | Tick collection methods and hosts |
| --- | --- | --- | --- | --- | --- |
| Adelson[1]; 2001 | *Bartonella* spp. | U: 37/107 | PCR, sequencing; controls; NG | NJ (Union) | Sweep |
|  | *Bb* | U: 36/107 |  |  |  |
|  | *Bam* | U: 9/107 |  |  |  |
|  | *Bartonella* spp. + *Bb* | U: 9/107 |  |  |  |
|  | *Ap* | U: 2/107 |  |  |  |
|  | *Bam* + *Bb* | U: 2/107 |  |  |  |
|  | *Ap + Bam + Bartonella* spp. | U: 1/107 |  |  |  |
|  | *Ap + Bartonella* spp. | U: 1/107 |  |  |  |
|  | *Ap + Bartonella* spp. *+ Bb* | U: 1/107 |  |  |  |
|  | *Bartonella* spp. *+ Bam* | U: 1/107 |  |  |  |
| Aliota[2]; 2011 | *Ap* | N: 23/67; A: 129/561 | PCR, sequencing, cell culture; controls; NA | NY (Dutchess, Putnam) | Flagging |
|  | *Bb* | N: 45/67; A: 338/561 |  |  |  |
|  | *Bam* | N: 13/67; A: 22/561 |  |  |  |
|  | *Ec* | N: 1/67; A: 11/561 |  |  |  |
|  | DTV | N: 0/67; A: 27/561 |  |  |  |
|  | *Ap + Bb* | A: 48/466 |  |  |  |
|  | *Bb + Bam* | A: 45/466 |  |  |  |
|  | *Bb* + DTV | A: 7/466 |  |  |  |
|  | *Bb + E. chaffeensis* | A: 2/466 |  |  |  |
|  | *Ap + Bb + Bam* | A: 7/466 |  |  |  |
|  | *Bb + Ec*+ DTV | A: 2/466 |  |  |  |
|  | *Ap + Bb* + DTV | A: 1/466 |  |  |  |
| Anderson[3]; 1994-2012 | DTV | A: 30/1911 | RT-PCR, sequencing, culture; positive controls; NA | CT (Fairfield, Hartford, New London, New Haven, Tolland, Windham) | Flagging; ex. deer |
|  | DTV | A: 2/2 |  | MA (Nantucket, Suffolk) |  |
|  | DTV | A: 5/5 |  | WI (Chippewa, Washburn) |  |
| Barbour[4]; 2002 | *Bb* | N: 244/689 | qPCR, sequencing; negative controls; NA | CT (unknown) | Flagging |
|  | *Bm* | N: 38/689 |  |  |  |
|  | *Bb + Bm* | N: 3/689 |  |  |  |
|  | *Bb* | N: 22/159 |  | MA (unknown) |  |
|  | *Bm* | N: 4/159 |  |  |  |
|  | *Bb* | N: 23/182 |  | IL (unknown) |  |
|  | *Bm* | N: 3/182 |  |  |  |
|  | *Bb* | N: 11/109 |  | IN (unknown) |  |
|  | *Bm* | N: 6/109 |  |  |  |
|  | *Bb* | N: 35/244 |  | MI (unknown) |  |
|  | *Bm* | N: 2/244 |  |  |  |
|  | *Bb* | N: 310/639 |  | MN (unknown) |  |
|  | *Bm* | N: 20/639 |  |  |  |
|  | *Bb* | N: 423/2529 |  | WI (unknown) |  |
|  | *Bm* | N: 52/2529 |  |  |  |
|  | *Bb* | N: 84/474 |  | MD (unknown) |  |
|  | *Bm* | N: 10/474 |  |  |  |
|  | *Bb* | N: 82/188 |  | ME (unknown) |  |
|  | *Bm* | N: 1/188 |  |  |  |
|  | *Bb* | N: 29/166 |  | NJ (unknown) |  |
|  | *Bm* | N: 8/166 |  |  |  |
|  | *Bb* | N: 269/1263 |  | NY(unknown) |  |
|  | *Bm* | N: 18/1263 |  |  |  |
|  | *Bb* | N: 70/297 |  | PA (unknown) |  |
|  | *Bm* | N: 4/297 |  |  |  |
|  | *Bb* | N: 22/94 |  | RI (unknown) |  |
|  | *Bm* | N: 1/94 |  |  |  |
|  | *Bb* | N: 30/173 |  | VA (unknown) |  |
|  | *Bm* | N: 6/173 |  |  |  |
|  | *Bb* | N: 17/89 |  | IA (unknown) |  |
|  | *Bm* | N: 1/89 |  |  |  |
| Benson[5]; 2002 | *Rickettsia* sp. IRS4 + *Burkholderia* sp. **+** *Delftia acidovorans* + *E. coli* + *Mycobacterium manitobense* + *Rhodococcus erythropolis* strain NVI + | N: 1/7 | PCR, sequencing, cloning; negative controls; GenBank | MA (Barnstable, Nantucket) | Flagging |
|  | *Rickettsia sp. IRS4 +* uncultured bacterium 1 + *Sphingomonas* sp. AV069 + *Delftia acidovorans* WDL34 + *E. coli* + *Rhodococcus erythropolis* strain NVI + *Propionibacterium acnes* | N: 1/7 |  |  |  |
|  | *Rickettsia sp. IRS4* + *Sphingomonas* sp. | N: 1/7 |  |  |  |
|  | *Wolbachia* sp. + uncultured bacterium 2 + uncultured bacterium 3 + *Sphingomonas elodea* + *Propionibacterium acnes* + *Bacillus* sp. + uncultured bacterium 6 + plastid cone + uncultured bacterium 9 | N: 1/7 |  |  |  |
|  | *Rickettsia sp. IRS4* + *Afipia felis* + *Sphingomonas elodea* + REIS + uncultured bacterium 12 | N: 1/7 |  |  |  |
|  | *Rickettsia sp. IRS4 + Ap + Sphingomonas melonis +* *Sphingopyxis alaskensis* + uncultured bacterium 4 + uncultured bacterium 5 + uncultured bacterium 7 + *Acinetobacter* sp. strain phenon 4 + Acinetobacter sp. 1 + Uncultured Streptococcus sp.+ uncultured bacterium 2 + uncultured bacterium 11 + uncultured bacterium 12 | N: 1/7 |  |  |  |
|  | *Rickettsia sp. IRS4 + Methylobacterium mesophilicum + Methylobacterium* sp. strain G296-5 *+ Sphingomonas* sp. strain BF2 *+ Stenotrophomonas maltophilia* + uncultured bacterium 6 + uncultured bacterium 8 + uncultured bacterium 13 | N: 1/7 |  |  |  |
| Bouchard[6]; 2007-08 | *Ap* | A: 36/243 | Multiplex qPCR, PCR, sequencing; no controls; NA | QC (Montérégie, Estrie) | Ex. white-tailed deer |
|  | *Bb* | A: 8/243 |  |  |  |
|  | *Bam* | A: 0/243 |  |  |  |
| Brackney[7]; 2007-08 | DTV | A: 17/1335 | Culture, RT-PCR, sequencing; no controls; NA | WI (Sawyer, Washburn) | Flagging |
| Cherepko[8]; 2004-06 | *Ap* | A: 17/443; N: 2/443 | PCR, sequencing; controls; NG | PA (Monroe) | Flagging; ex. humans, domestic animals |
|  | *Bb* | A: 21/443; N: 6/443 |  |  |  |
|  | *Barh* | A: 14/272; N: 3/272 |  |  |  |
|  | *Bam* | A: 3/443; N: 0/443 |  |  |  |
|  | *Bartonella* sp. | A: 7/272; N: 2/272 |  |  |  |
|  | *Barh + Ap* | A: 2/272; N: 0/272 |  |  |  |
|  | *Barh + Bb* | A: 2/272; N: 0/272 |  |  |  |
|  | *Bb + Ap* | A: 9/443; N: 0/443 |  |  |  |
|  | *Ap + Bam* | A: 1/443; N: 0/443 |  |  |  |
|  | *Bb + Ap + Bar. henselae* | A:1/272; N: 0/272 |  |  |  |
|  | *Bb + Ap + Bartonella* spp. | A: 3/272; N: 0/272 |  |  |  |
| Cohen[9]; 2007-08 | *Ec* | U: 0/4 | Nested PCR, sequencing, positive controls; NA | TN (>15 counties) | Dragging; ex. humans, wild animals |
|  | *E. ewingii* | U: 0/4 |  |  |  |
| Courtney[10]; 2000-01 | *Ap* | A: 81/454 | Nested PCR, sequencing; no controls; NA | PA (Chester, Delaware, Erie) | Flagging; ex. white-tailed deer |
|  | *Bb* | A: 187/454 |  |  |  |
|  | *Ap + Bb* | A: 16/454 |  |  |  |
| Crowder[11]; 2008-12 | *Bm* | A/N: 16/369 | PCR/ESI-MS, sequencing, mass-spec; controls; NA | CT (Fairfield, Litchfield, New London) | Flagging |
|  | *Bm* | A/N: 10/81 |  | IN (Pulaski) |  |
|  | *Bm* | A/N: 7/581 |  | NY (Dutchess, Suffolk, Westchester) |  |
|  | *Bm* | A/N: 2/80 |  | PA (Chester) |  |
| Curran[12]; 1998 | *Ap* | U: 2/150 | Nested PCR; controls; NA | DE (Kent, New Castle, Sussex) | Ex. white-tailed deer |
|  | *Bb* | U: 14/150 |  |  |  |
| Dibernardo[13]; 2012 | *Ap* | L/N/A: 5/87 | Duplex PCR, PCR-ESI-MS, PCR, sequencing; controls; NA | AB (unknown) | Passive surveillance |
|  | *Bb* | L/N/A: 12/87 |  |  |  |
|  | *Bm* | L/N/A: 1/87 |  |  |  |
|  | *Ap + Bb* | L/N/A: 1/87 |  |  |  |
|  | *Ap* | L/N/A: 7/170 |  | MB (unknown) |  |
|  | *Bb* | L/N/A: 15/170 |  |  |  |
|  | *Bm* | L/N/A: 2/170 |  |  |  |
|  | *Ap + Bb* | L/N/A: 3/170 |  |  |  |
|  | *Ap* | L/N/A: 3/366 |  | NB (unknown) |  |
|  | *Bb* | L/N/A: 25/366 |  |  |  |
|  | *Bm* | L/N/A: 3/366 |  |  |  |
|  | *Ap + Bb* | L/N/A: 1/366 |  |  |  |
|  | *Ap* | L/N/A: 1/33 |  | NL (unknown) |  |
|  | *Bb* | L/N/A: 9/33 |  |  |  |
|  | *Ap + Bb* | L/N/A: 1/33 |  |  |  |
|  | *Bm* | L/N/A: 0/33 |  |  |  |
|  | *Bb* | L/N/A: 4/34 |  | NS (unknown) |  |
|  | *Bm* | L/N/A: 1/34 |  |  |  |
|  | *Bm + Bb* | L/N/A: 1/34 |  |  |  |
|  | *Ap* | L/N/A: 0/34 |  |  |  |
|  | *Ap* | L/N/A: 7/2591 |  | ON (unknown) |  |
|  | *Bb* | L/N/A: 411/2591 |  |  |  |
|  | *Bm* | L/N/A: 7/2591 |  |  |  |
|  | *Ap + Bb* | L/N/A: 2/2591 |  |  |  |
|  | *Bm + Bb* | L/N/A: 2/2591 |  |  |  |
|  | *Ap* | L/N/A: 1/178 |  | PE (unknown) |  |
|  | *Bb* | L/N/A: 17/178 |  |  |  |
|  | *Bm* | L/N/A: 1/178 |  |  |  |
|  | *Ap* | L/N/A: 18/1479 |  | QC (unknown) |  |
|  | *Bb* | L/N/A: 203/1479 |  |  |  |
|  | *Bm* | L/N/A: 8/1479 |  |  |  |
|  | *Ap + Bb* | L/N/A: 3/1479 |  |  |  |
|  | *Bm + Bb* | L/N/A: 5/1479 |  |  |  |
| Drebot[14]; 1999 | *Ap* | A: 1/120 | Nested PCR, sequencing; no controls; NA | ON (Haldimand-Norfolk) | Ex. white-tailed deer |
| Diuk-Wasser[15]; 2007, 2010 | *Bam* | N: 80/1083 | Nested PCR, qPCR; no controls; NA | CT (New London, Tolland, Windham) | Dragging |
|  | *Bb* | N: 253/1083 |  |  |  |
|  | *Bb + Bam* | N: 36/1083 |  |  |  |
|  | *Bam* | N: 8/87 |  | MA (Nantucket) |  |
|  | *Bb* | N: 18/87 |  |  |  |
|  | *Bb + Bam* | N: 1/87 |  |  |  |
| Dupuis[16]; 2007-12 | DTV | A/N: 43/1153 | Culture, RT-qPCR, sequencing; no controls; NA | NY (Dutchess, Putnam, Westchester, Rockland, Orange, Ulster, Sullivan) | Flagging; ex. multiple mammal and bird species |
| Ebel[17]; 1996-99 | DTV | A: 2/287 | RT-PCR, sequencing; positive control; NA | MA (Barnstable, Nantucket) | Ex vegetation |
|  | DTV | A: 1/180 |  | RI (Newport) |  |
|  | DTV | A: 10/792 |  | WI (Washburn) |  |
| Eskow[18]; “2001” | *Barh* + *Bb* | U: 1/1 | PCR, sequencing; controls; NA | NJ “Central” | Ex. patient home, cat |
|  | *Barh* | U: 1/? |  |  |  |
|  | *Bb* | U: 0/? |  |  |  |
|  | *Babesia* spp. | U: 0/? |  |  |  |
|  | *Ehrlichia* spp. | U: 0/? |  |  |  |
| Fang[19]; “2002” | *Ap* | A: 0/248 | Nested PCR, sequencing; controls; NA | FL (Duval, Nassau, St. Johns) | Flagging |
|  | *Ap* | A: 7/132 |  | GA (Chatham, Emanuel, Glynn, Jenkins, McIntosh) |  |
|  | *Ap* | A: 6/438 |  | SC (Beauford, Colleton, Jasper, Orangeburg) |  |
| Fritzen[20]; 2009-10 | *Bad* | U: 7/166 | PCR, sequencing; controls; NA | TN (unknown) | Dragging; ex. white-tailed deer, dog |
|  | *Theileria cervi* | U: 24/166 |  |  |  |
| Goltz[21]; 2010-11 | *Bad* | A: 4/244 | Nested PCR, sequencing; controls; NA | MS (Marshall, Oktibbeha) | Flagging |
|  | *Bb* | A: 0/244 |  |  |  |
|  | *Ap* | A: 0/244 |  |  |  |
| Grant-Klein[22]; 2008-09 | DTV | A: 3/322 | Culture, RT-PCR, sequencing, mass spec; positive control; NA | CT (Bridgeport) | Flagging |
|  | POWV | A: 0/322 |  |  |  |
|  | DTV | A: 5/322 |  | NY (Westchester, Suffolk) |  |
|  | POWV | A: 8/322 |  |  |  |
| Hamer[23]; 2004-07 | *Bb* | L: 0/2; N: 1/6 pools | Nested PCR, sequencing; positive controls; NA | MI (Kalamazoo) | Ex. rabbits, birds |
|  | *B. andersonii* | L: 1/2; N: 0/6 pools |  |  |  |
|  | *Bm* | L: 0/2; N: 0/6 pools |  |  |  |
| Hamer[24]; 2005 | *Bb* | A/N: 3/18 | PCR, sequencing; positive controls; NA | MI (>15 counties) | Ex. dogs |
|  | *Bam* | A/N: 0/18 |  |  |  |
|  | *Ap* | A/N: 0/18 |  |  |  |
| Hamer[25]; 2006 | *Bb* | A: 60/119; N: 3/11 | PCR, sequencing; no controls; NA | MI (Menominee) | Flagging |
|  | *Ap* | A: 5/119; N: 2/11 |  |  |  |
|  | *Bad* | A: 6/119; N: 0/11 |  |  |  |
|  | *Bb + Ap* | A: 2/119; N: 1/11 |  |  |  |
|  | *Bb + Bad* | A: 5/119; N: 0/11 |  |  |  |
|  | *Bl* | A: 0/119; N: 0/11 |  |  |  |
|  | *Bm* | A: 0/119; N: 0/11 |  |  |  |
|  | *Bam* | A: 0/119; N: 0/11 |  |  |  |
| Hamer[26]; 2006-07 | *Ap* | A: 9/61 | qPCR, sequencing; controls; NA | MN (Pine) | Flagging |
|  | *Bb* | A: 52/61 |  |  |  |
|  | *Bm* | A: 0/61 |  |  |  |
|  | *Bad* | A: 0/61 |  |  |  |
|  | *Bam* | A: 1/61 |  |  |  |
|  | *Ap* | A: 18/253 |  | IL (Cook, Ogle) |  |
|  | *Bb* | A: 123/253 |  |  |  |
|  | *Bm* | A: 4/253 |  |  |  |
|  | *Bad* | A: 14/253 |  |  |  |
|  | *Bam* | A: 0/253 |  |  |  |
|  | *Ap* | A: 19/297 |  | IN (Porter, Pulaski) |  |
|  | *Bb* | A: 146/297 |  |  |  |
|  | *Bm* | A: 4/297 |  |  |  |
|  | *Bad* | A: 11/297 |  |  |  |
|  | *Bam* | A: 0/297 |  |  |  |
|  | *Ap* | A: 28/296 |  | MI (Allegan, Menominee, Van Buren) |  |
|  | *Bb* | A: 173/296 |  |  |  |
|  | *Bm* | A: 1/296 |  |  |  |
|  | *Bad* | A: 18/296 |  |  |  |
|  | *Bam* | A: 0/296 |  |  |  |
|  | *Ap* | A: 67/622 |  | WI (Iowa, Monroe) |  |
|  | *Bb* | A: 309/622 |  |  |  |
|  | *Bm* | A: 25/622 |  |  |  |
|  | *Bad* | A: 27/622 |  |  |  |
|  | *Bam* | A: 4/622 |  |  |  |
| Hamer[27]; 2005-10 | *Ap* | L: 0/6 pools (n=22); N: 0/6 pools (n=?) | qPCR, sequencing; no controls; NA | IL (Cook) | Ex. multiple bird species |
|  | *Bb* | L: 1/6 pools (n=22); N: 3/6 pools (n=?) |  |  |  |
| Hamer[28]; 2010 | *Ap* | L: 0/5; N=0/8 | qPCR, sequencing; controls; NA | IL (Cook) | Ex. multiple bird species |
|  | *Bb* | L: 1/5; N: 5/8 |  |  |  |
| Han[29]; 2009 | *Ap* | A: 0/15; N: 0/110 | PCR; no controls; NA | PA (Adams) | Dragging |
|  | *Bb* | A: 13/15; N: 74/110 |  |  |  |
|  | *Bam* | A: 0/15; N: 0/110 |  |  |  |
| Herrin[30]; 2012-13 | *Ap* | A: 3/356 | PCR, sequencing; no controls; NA | VA (Giles, Pulaski) | Dragging |
|  | *Bb* | A: 117/356 |  |  |  |
|  | *Ap + Bb* | A: 2/356 |  |  |  |
| Hersh[31]; 2011-12 | *Ap* | N: 445/7643 | Multiplex qPCR, sequencing; no controls; NA | NY (Dutchess) | Flagging; ex. multiple mammal and bird species |
|  | *Bam* | N: 499/7643 |  |  |  |
|  | *Bb* | N: 1765/7643 |  |  |  |
|  | *Ap + Bam* | N: 39/7643 |  |  |  |
|  | *Ap + Bb* | N: 223/7643 |  |  |  |
|  | *Bam + Bb* | N: 708/7643 |  |  |  |
|  | *Bam + Bb + Ap* | N: 46/7643 |  |  |  |
| Hersh[32]; 2008-10 | *Bam* | N: 149/4306 | qPCR, sequencing; controls; NA | NY (Dutchess) | Dragging; ex. multiple mammal and bird species |
| Hoen[33]; 1998-2003 | *Ap* | A: 155/1828; N: 180/4276 | Nested PCR, sequencing; controls; NA | CT (New London); MD (Anne Arundel, Baltimore, Prince George); NJ (Monmouth); NY (Dutchess, Orange, Putnam, Rockland, Ulster, Westchester); RI (Washington) | Dragging; flagging |
|  | *Bb* | A: 698/1828; N: 229/4276 |  |  |  |
|  | *Bm* | A: 35/1828; N: 34/4276 |  |  |  |
|  | *Bb + Ap* | A: 23/711; N: 2/445 |  |  |  |
|  | *Bb + Bm* | A: 8/479; N: 1/178 |  |  |  |
|  | *Ap + Bm* | A: 0/103; N: ?/47 |  |  |  |
| Holman[34]; 1995-97 | *Ap* | A/N: 11/374 | Fluorescent microscopy, PCR, sequencing; controls; NA | ME (Hancock, Knox, Lincoln, York, Waldo) | Ex. white-footed mouse, Eastern chipmunk, pets |
|  | *Bad* | A/N: 19/374 |  |  |  |
|  | *Bam* | A/N: 3/374 |  |  |  |
|  | *Bb* | A/N: 88/374 |  |  |  |
|  | *Bb + Ap* | A/N: 2/374 |  |  |  |
|  | *Bb + Bam* | A/N: 2/374 |  |  |  |
| Keesing[35]; 2007-10 | *Ap* | U: 345/4152; N: 44/890 | PCR, sequencing; controls; NA | NY (Dutchess) | Dragging, ex. mammals, birds |
| Kogut[36]; 2001 | *Bam* | A/N: 5/123 pools (n=1139) | PCR (blind), sequencing; negative controls; NA | NY (Columbia, Dutchess, Putnam, Westchester) | Flagging |
| Krakowetz[37]; 2007-10 | *Ap* | U: 3/89 | qPCR, PCR-RFLP, TaqMAN SNP Assay, sequencing; no controls; NA | AB (unknown) | Passive surveillance; dragging |
|  | *Ap* | U: 32/570 |  | MB (Pembina Valley Provincial Park, Stanley Trail, +others) |  |
|  | *Ap* | U: 15/763 |  | NB (unknown) |  |
|  | *Ap* | U: 2/62 |  | NL (unknown) |  |
|  | *Ap* | U: 19/1649 |  | NS (unknown) |  |
|  | *Ap* | U: 13/4407 |  | ON (Leeds-Grenville-Lanark, Niagara, +others) |  |
|  | *Ap* | U: 10/359 |  | PE (unknown) |  |
|  | *Ap* | U: 71/4697 |  | QC (unknown) |  |
|  | *Ap* | U: 4/10 |  | SK (unknown) |  |
|  | *Ap* | U: ?/168 |  | MN (Becker, Clearwater, Hubbard, Morrison, Pine) |  |
| Kurtti[38]; 2007 | *R. buchneri* | A: 1/1 | PCR, sequencing, culture, TEM; no controls; GenBank | MN (Anoka) | Ex. dog |
| Layfield[39]; 1998-99 | *Ap* | U: 4/103 | Nested PCR, sequencing; controls; NA | MN (Billings, Eddy, Grand Forks, McKenzie, Morton, Pembina, Ramsey, Rolette, Steele) | Ex. dogs, human, cats, horses, deer |
|  | *Bb* | U: 17/103 |  |  |  |
|  | *Ap + Bb* | U: 1/103 |  |  |  |
| Lee[40]; 2009-13 | *Ap* | N: 66/1214 | Nested PCR, sequencing, no controls; NA | WI (Adams, Clark, Fond du Lac, Iowa, Jackson, La Crosse, Marquette, Portage, Sauk, Sheboygan, Walworth, Waupaca, Waushara, Washington, Wood) | Dragging |
|  | *Bb* | N: 317/1190 |  |  |  |
|  | *Bb + Ap* | N: 22/1190 |  |  |  |
| Leydet[41]; 2010-11; | *R. parkeri* | A: 3/18 | PCR, sequencing, cloning; no controls; NA | LA (Concordia, Iberville,  Livingston, Madison, Pointe Coupee, St. Landry, St. Mary) | Ex. black bears |
|  | *Bb* | A: 2/18 |  |  |  |
|  | REIS | A: 7/18 |  |  |  |
|  | *Ap* | A: 0/18 |  |  |  |
|  | *Bam* | A: 0/18 |  |  |  |
|  | *Ehrlichia* spp. | A: 0/18 |  |  |  |
|  | *B. bissettii* | A: 1/18 |  |  |  |
|  | *Bb +* REIS | A: 1/18 |  |  |  |
|  | REIS *+ R. parkeri* | A: 1/18 |  |  |  |
| Lovrich[42]; 2008 | *Ap* | A: 24/201 | qPCR, sequencing; controls; NA | WI (Trempealeau, Vernon) | Flagging |
| Margos[43]; 1987, 2007 | *Bk* | A: 4/4 | Culture, sequencing, RFLP; no controls; NA | NY (Dutchess) | Ex. white-footed mouse, human |
|  | *Bk* | A: 1/1 |  | NS (unknown) |  |
| Massung[44]; 1996-99 | *Ap* | A/N: 59/454 | Nested PCR, sequencing; no controls; NA | CT (Fairfield, New London) | “questing ticks collected” |
|  | *Ap* | A/N: 123/538 |  | RI (Washington) |  |
| Massung[45]; 1996-99 | *Ap* | N: 16/116 | Nested PCR, sequencing; no controls; NA | RI (Washington) | Unknown |
| Mays[46]; 2011-12 | *R. amblyommii* | A: 1/47 | Nested PCR, qPCR, sequencing; controls; GenBank | TN (Fayette, Hardeman) | Ex. white-tailed deer |
|  | Unidentified *Rickettsia* sp. | A: 2/47 |  |  |  |
|  | REIS | A: 24/47 |  |  |  |
|  | *Ap* | A: 1/47 |  |  |  |
|  | *E. ewingii* | A: 2/47 |  |  |  |
|  | *Bb* | A: 0/47 |  |  |  |
|  | *Babesia* spp. | A: 0/47 |  |  |  |
|  | *Ehrlichia* Panola Mountain sp. | A: 1/47 |  |  |  |
| McCall[47]; 2008 | *Ap* | A: 7/30 | Nested PCR; no controls; NA | RI (Washington) | Unknown |
|  | *Bb* | A: 20/30 |  |  |  |
| Michalski[48]; “2006” | *Ap* | U: 17/171 | Nested PCR, sequencing; positive controls; NA | WI (Buffalo, Green Lake, Waupaca, Waushara) | Flagging, ex. deer, dogs |
| Moreno[49]; 2003-04 | Unidentified *Rickettsia* sp. (likely REIS) | A/N: 1/147 (49 pools) | PCR, TTGE, sequencing; controls; GenBank | NY (Dutchess, Westchester) | Flagging, ex. white-tailed deer |
|  | *Ap* | A/N: 1/147 (49 pools) |  |  |  |
|  | Unidentified *Borrelia* sp. | A/N: 1/147 (49 pools) |  |  |  |
|  | *Afipia broomeae* | A/N: 1/147 (49 pools) |  |  |  |
|  | *Sphingobacterium* sp. | A/N: 1/147 (49 pools) |  |  |  |
|  | *Ralstonia mannitolilytica* | A/N: 1/147 (49 pools) |  |  |  |
|  | *Enterobacter asburiae* | A/N: 1/147 (49 pools) |  |  |  |
|  | *Photorhabdus* sp. | A/N: 1/147 (49 pools) |  |  |  |
|  | *Raoultella* sp. | A/N: 1/147 (49 pools) |  |  |  |
|  | *Shigella* sp. | A/N: 1/147 (49 pools) |  |  |  |
|  | Uncultured gamma protebacterium | A/N: 1/147 (49 pools) |  |  |  |
|  | *Acenetobacter* sp. 2 | A/N: 1/147 (49 pools) |  |  |  |
|  | *Pseudomonas fluorescens* | A/N: 1/147 (49 pools) |  |  |  |
|  | *Pseudomonas* sp. 1 | A/N: 1/147 (49 pools) |  |  |  |
|  | *Pseudomonas* sp. 2 | A/N: 1/147 (49 pools) |  |  |  |
|  | *Pseudomonas* sp. 3 | A/N: 1/147 (49 pools) |  |  |  |
|  | Symbiont cf. *Pseudomonas* | A/N: 1/147 (49 pools) |  |  |  |
|  | Uncultured *Stenotrophomonas* sp. | A/N: 1/147 (49 pools) |  |  |  |
|  | *Stenotrophomonas* sp. | A/N: 1/147 (49 pools) |  |  |  |
|  | *Rhodococcus* sp. | A/N: 1/147 (49 pools) |  |  |  |
|  | *Williamsia* sp. | A/N: 1/147 (49 pools) |  |  |  |
|  | Uncultured bacterium 14 | A/N: 1/147 (49 pools) |  |  |  |
|  | Uncultured bacterium 15 | A/N: 1/147 (49 pools) |  |  |  |
| Moncayo[50]; 2007-08 | *R. cooleyi* | A; 2/2; N 2/4 | qPCR, RFLP, sequencing; controls; NA | TN (>15 counties) | Ex. humans, wild animals; dragging |
| Nelder[51]; 2008-12 | *Ap* | A/N: 18/6046; | Multiplex qPCR, sequencing; no controls; NA | ON (>15 health units) | Ex. humans |
|  | *Bb* | A: 873/5763; N: 32/283 |  |  |  |
| Ogden[52]; 2005-06 | *Ap* | N: 3/209; L: 0/53 | Nested PCR, qPCR, RLB, sequencing; no controls; NA | ON (Haldimand-Norfolk, Hastings Prince Edward County, Windsor-Essex, Thunder Bay District, Toronto) | Ex. birds |
|  | *Bb* | N: 32/209; L: 0/53 |  |  |  |
|  | *Ap* | N: 0/4 |  | NS (Southwest Health) |  |
|  | *Bb* | N: 0/4 |  |  |  |
| Ogden[53]; 2005-07 | *Bb* | A: 1/1 | PCR, MLST typing, sequencing; no controls; NA | AB (unknown) | Ex. companion animals and humans |
|  | *B. kurtenbachii* | A: 0/1 |  |  |  |
|  | *Bm* | A: 0/1 |  |  |  |
|  | *Bb* | A: 6/6 |  | MB (unknown) |  |
|  | *B. kurtenbachii* | A: 0/6 |  |  |  |
|  | *Bm* | A: 0/6 |  |  |  |
|  | *Bb* | A: 64/67 |  | ON (unknown) |  |
|  | *B. kurtenbachii* | A: 0/67 |  |  |  |
|  | *Bm* | A: 3/67 |  |  |  |
|  | *Bb* | A: 31/31 |  | QC (unknown) |  |
|  | *B. kurtenbachii* | A: 0/31 |  |  |  |
|  | *Bm* | A: 0/31 |  |  |  |
|  | *Bb* | A: 13/13 |  | NB (unknown) |  |
|  | *Bk* | A: 0/13 |  |  |  |
|  | *Bm* | A: 0/13 |  |  |  |
|  | *Bb* | A: 20/24 |  | NS (unknown) |  |
|  | *Bk* | A: 1/24 |  |  |  |
|  | *Bm* | A: 3/24 |  |  |  |
|  | *Bb* | A: 1/1 |  | NL (unknown) |  |
|  | *Bk* | A: 0/1 |  |  |  |
|  | *Bm* | A: 0/1 |  |  |  |
|  | *Bb* | A: 22/22 |  | PE (unknown) |  |
|  | *Bk* | A: 0/22 |  |  |  |
|  | *Bm* | A: 0/22 |  |  |  |
| Pritt[54]; 2009 | *Ehrlichia* sp. (nr. *muris*) | N: 7/202; A: 9/332 | qPCR, nested PCR, sequencing; controls; GenBank | MN (Morrison, Pine) | Flagging |
|  | *Ehrlichia* sp. (nr. *muris*) | N: 1/6 pool (n=154) |  | WI (Eau Claire) |  |
| Pruisinski[55]; 2003-06 | *Ap* | A: 973/7904  N: 213/3300 | Multiplex PCR, sequencing; controls; NA | NY (Columbia, Dutchess, Putnam, Westchester, Greene, Ulster, Orange, Rockland) | Flagging |
|  | *Bam* | A: 89/7904; N: 194/3300 |  |  |  |
|  | *Bb* | A: 3610/7904; N: 474/3300 |  |  |  |
|  | *Bam + Ap* | N: 1/3300; A: 12/7904 |  |  |  |
|  | *Bb + Ap* | A: 53/7904; N: 15/3300 |  |  |  |
|  | *Bb* + *Bam* | N: 12/3300; A: 28/7904 |  |  |  |
|  | *Bb + Bam + Ap* | A: 12/7904; N: 0/3300 |  |  |  |
| Roellig[56]; 2004-05 | *Ap* | A: 150/765 | Nested PCR, sequencing; no controls; NA | GA (Bulloch, Camden, Chatham, Cumberland, Glynn) | Dragging |
| Rollend[57]; 2010 | *Bam* | N: 0/115 | qPCR, sequencing; controls; NA | CT (Mansfield, Salisbury) | Dragging |
|  | *Bam* | N: 37/764 |  | MA (Nantucket) |  |
| Russart[58]; 2010 | *Ap* | A: 6/34; N:2/45; L: 0/15 | PCR, sequencing; no controls; NA | ND (Eddy, Grand Forks, Pembina, Ramsey, Rolette, Steele) | Flagging, ex. multiple mammal species, humans |
|  | *Bb* | A: 2/32; N: 1/45; L: 0/15 |  |  |  |
|  | *Ap + Bb* | N: 1/94 |  |  |  |
|  | *Bam* | A/N/L: 0/? |  |  |  |
| Schulze[59]; 2001-05 | *Bb* | A: 30/94 | PCR, sequencing; positive controls; NA | NJ (Monmouth) | Dragging; ex. humans |
| Schulze[60]; 2003-04 | *Ap* | A: 9/147 | PCR, sequencing; controls; NG | NJ (Monmouth) | Dragging; walk |
|  | *Bb* | A: 74/147 |  |  |  |
|  | *Borrelia* n. sp. (likely *Bm*) | A: 2/147 |  |  |  |
|  | *Bb + Ap* | A: 4/147 |  |  |  |
|  | *Bb +* novel *Borrelia* | A: 1/147 |  |  |  |
|  | *Ap +* novel *Borrelia* | A: 1/147 |  |  |  |
| Schulze[61]; 2004-07 | *Bam* | A: 50/610; N: 19/478 | PCR, sequencing; positive controls; NA | NJ (Monmouth) | Dragging, walk; ex. humans |
|  | *Bb* | A: 276/610; N: 48/478 |  |  |  |
|  | *Bb + Bam* | A: 38/610; N: 14/478 |  |  |  |
| Scoles[62]; 1999 | *Bb* | N: 0/162 | PCR, sequencing; controls; NG | MD (Baltimore) | Flagging; dragging |
|  | Novel *Borrelia* (likely *Bm*) | N: 0/162 |  |  |  |
|  | *Bb* | N: 17/160 |  | NY (Westchester) |  |
|  | Novel *Borrelia* (likely *Bm*) | N: 4/160 |  |  |  |
|  | *Bb* | N: 32/168 |  | CT (New London) |  |
|  | Novel *Borrelia* (likely *Bm*) | N: 4/168 |  |  |  |
|  | *Bb* + *Bm* | N: 1/168 |  |  |  |
|  | *Bb* | N: 45/182 |  | RI (Washington) |  |
|  | Novel *Borrelia* (likely *Bm*) | N: 3/182 |  |  |  |
|  | *Bb* | N: 18/202 |  | NJ (Monmouth) |  |
|  | Novel *Borrelia* (likely *Bm*) | N: 4/202 |  |  |  |
| Shukla[63]; 1998 | *Ap* | A/N: 24/636 | PCR, Southern hybridization, sequencing; no controls; NA | WI (unknown) | Flagging |
|  | REIS | A/N: 11/636 |  |  |  |
| Smith[64]; 1995-2011 | *Bam* | A: 7/936 | PCR, fluorescent microscopy, sequencing; no controls; NA | ME (Androscoggin, Cumberland, Hancock, Knox, Lincoln, Oxford, Penobscot, Piscataquis, Sagadahoc, Somerset, York, Waldo, Washington) |  |
|  | *Bad* | A: 0/936 |  |  |  |
|  | *Bb* | A: 4688/9342 |  |  |  |
| Smith[65]; 2006 | *Bb* | A: 0/4; N: 6/15 | qPCR, sequencing; controls; NG | NC (Chatham) | Flagging |
|  | *R. massiliae/Rickettsia* sp. Bar 29 | A: 0/4; N: 3/19 |  |  |  |
|  | Unidentified *Rickettsia* sp. | A/N: 1/19 |  |  |  |
|  | *Ehrlichia* sp. | A: 0/4; N: 0/15 |  |  |  |
| Steiner[66]; 2003-04 | *Ap* | A: 52/276; N: 1/39 pools (n=117) | PCR, sequencing, restriction enzyme; no controls; NA | IN (Pulaski) | Dragging; ex. deer, dogs |
|  | *Bad* | A: 19/276; N: 0/39 pools (n=117) |  |  |  |
|  | *Bad + Ap* | A: 6/276 |  |  |  |
| Steiner[67]; 2003-04 | *Ap* | A: 5/100 | PCR, sequencing; controls; NA | IN (Pulaski) | Dragging |
|  | *Bb* | A: 72/100 |  |  |  |
|  | *Bad* | A: 6/100 |  |  |  |
|  | REIS | A: 63/100 |  |  |  |
|  | *Ap + Bb* | A: 4/100 |  |  |  |
|  | *Bb + Babesia* sp. | A: 4/100 |  |  |  |
|  | *Bb +* REIS | A: 41/100 |  |  |  |
|  | *Ap +* REIS | A: 3/100 |  |  |  |
|  | *Babesia* spp. *+* REIS | A: 5/100 |  |  |  |
|  | *Ap* | A: 16/100 |  | ME (York) |  |
|  | *Bb* | A: 58/100 |  |  |  |
|  | *Bam* | A: 7/100 |  |  |  |
|  | *Bad* | A: 15/100 |  |  |  |
|  | REIS | A: 46/100 |  |  |  |
|  | *Ap + Bb* | A: 9/100 |  |  |  |
|  | *Bb + Babesia* sp. | A: 11/100 |  |  |  |
|  | *Bb +* REIS | A: 21/100 |  |  |  |
|  | *Ap + Babesia* sp. | A: 2/100 |  |  |  |
|  | *Ap +* REIS | A: 8/100 |  |  |  |
|  | *Babesia* spp*. +* REIS | A: 10/100 |  |  |  |
|  | *Ap* | A: 1/94 |  | PA (Presque Isle) |  |
|  | *Bb* | A: 52/94 |  |  |  |
|  | *Bad* | A: 2/94 |  |  |  |
|  | REIS | A: 61/94 |  |  |  |
|  | *Ap + Bb* | A: 1/94 |  |  |  |
|  | *Bb + Babesia* sp. | A: 1/94 |  |  |  |
|  | *Bb +* REIS | A: 30/94 |  |  |  |
|  | *Ap +* REIS | A: 1/94 |  |  |  |
|  | *Babesia* spp*. +* REIS | A: 2/94 |  |  |  |
|  | *Ap* | A: 14/100 |  | WI (Monroe) |  |
|  | *Bb* | A: 35/100 |  |  |  |
|  | *Bad* | A: 11/94 |  |  |  |
|  | REIS | A: 82/94 |  |  |  |
|  | *Ap + Bb* | A: 8/100 |  |  |  |
|  | *Bb + Babesia* sp. | A: 4/100 |  |  |  |
|  | *Bb +* REIS | A: 29/100 |  |  |  |
|  | *Ap + Babesia* sp*.* | A: 1/100 |  |  |  |
|  | *Ap +* REIS | A: 14/100 |  |  |  |
|  | *Babesia* sp. *+* REIS | A: 8/100 |  |  |  |
| Swanson[68]; 2003 | *Ap* | N: 1/348 | Semi-nested PCR, sequencing; controls; GenBank | MD (Caroline, Dorchester, Kent, Queen Anne’s, Somerset, Talbot, Wicomico, Worcester) | Flagging |
|  | *Bb* | N: 51/348 |  |  |  |
|  | Unidentified *Bartonella* sp. | N: 3/348 |  |  |  |
|  | Unidentified *Rickettsia* sp. | N: 1/348 |  |  |  |
| Taft[69]; 1997-2000 | *Bb* | A/N: 0/2 | PCR, dot hybridization; sequencing; negative controls; NA | KY (Bullitt, Hardin) | CO^2^ traps; ex. humans, white-tailed deer, white-footed mouse |
|  | *Bl* | A/N: 0/2 |  |  |  |
|  | *Bb* | A/N: 0/56 |  | MA (Barnstable) |  |
|  | *Bl* | A/N: 1/56 |  |  |  |
|  | *Bb* | A/N: 5/39 |  | MD (Charles, Montgomery) |  |
|  | *Bl* | A/N: 0/39 |  |  |  |
|  | *Bb* | A/N: 0/4 |  | NC (Cumberland) |  |
|  | *Bl* | A/N: 0/4 |  |  |  |
|  | *Bb* | A/N: 3/68 |  | NJ (Burlington) |  |
|  | *Bl* | A/N: 0/68 |  |  |  |
|  | *Bb* | A/N: 1/31 |  | NY (Orange) |  |
|  | *Bl* | A/N: 1/31 |  |  |  |
|  | *Bb* | A/N: 0/47 |  | PA (Franklin, Lebanon) |  |
|  | *Bl* | A/N: 0/47 |  |  |  |
|  | *Bb* | A/N: 8/32 |  | VA (Brunswick, Caroline, Dinwiddie, Fairfax, James City, Nottoway, Prince George, York) |  |
|  | *Bl* | A/N: 0/32 |  |  |  |
| Telford[70]; 1992-97 | *E. muris* | A: 7/760 | Nested PCR, sequencing; no controls; NA | WI (Washburn) | Sweeping vegetation |
|  | *Ap* | A: 3/670 |  |  |  |
| Tokarz[71]; 2006-07 | *Bb* | A: 51/88 | Multiplex PCR, sequencing; controls; NG | NY (Suffolk) | Unknown |
|  | *Bl/miyamotoi* | A: 4/88 |  |  |  |
|  | *Barh* | A: 2/88 |  |  |  |
|  | *Ap* | A: 14/88 |  |  |  |
|  | *Bam* | A: 5/88 |  |  |  |
|  | *C. burnetii* | A: 0/88 |  |  |  |
|  | *Ec* | A: 0/88 |  |  |  |
|  | *F. tularensis* | A: 0/88 |  |  |  |
|  | *R. rickettsii* | A: 0/88 |  |  |  |
|  | *Ap + Bb* | A: 4/88 |  |  |  |
|  | *Bb + Bam* | A: 1/88 |  |  |  |
|  | *Bb + B miyamotoi* | A: 1/88 |  |  |  |
|  | *Ap* + *Bb* + *Bam* | A: 1/88 |  |  |  |
|  | *Ap* + *Bb* + *Ba. miyamotoi* | A: 1/88 |  |  |  |
| Tokarz[72]; 2008 | *Ap* | A: 56/286 | MasTAQ PCR, sequencing; no controls; NA | NY (Suffolk, Westchester) | Ex. vegetation |
|  | *Bb* | A: 182/286 |  |  |  |
|  | *Bm* | A: 7/286 |  |  |  |
|  | *Bam* | A: 58/286 |  |  |  |
|  | DTV | A: 7/286 |  |  |  |
|  | *Ap + Bb* | A: 45/286 |  |  |  |
|  | *Bam + Bb* | A: 48/286 |  |  |  |
|  | *Ap + Bam + Bb* | A: 14/286 |  |  |  |
|  | *Ap + Bm +. B burgdorferi* | A: 1/286 |  |  |  |
|  | *F. tularensis* | A: 0/286 |  |  |  |
|  | *Bl* | A: 0/286 |  |  |  |
|  | POWV *+ Ap* | A: 1/286 |  |  |  |
|  | POWV *+ Bb* | A: 7/286 |  |  |  |
|  | *Ap + Bam + Bb + Bm* | A: 1/286 |  |  |  |
| Tokarz[73]; 2013 | South Bay virus | A: 2/2 pools (n=60) | High-throughput sequencing; no controls; NG | NY (Suffolk) | Unknown |
|  | Blacklegged tick phlebovirus | A: 2/2 pools (n=60) |  |  |  |
|  | DTV | A: 1/2 pools (n=60) |  |  |  |
|  | *Ixodes scapularis* mononegavirales | A: 1/2 pools (n=60) |  |  |  |
| Trout-Fryxell[74]; 2006-08 | *Bb* | L: 0/1; N: 0/13; A: 12/678 | PCR, sequencing; controls; NA | AR (>15 counties) | Ex. dogs, deer |
|  | *Bl* | L: 0/1; N: 2/13; A: 53/678 |  |  |  |
| Ullmann[75]; 2003 | *Bb* | N: 44/250 | Multiplex PCR, sequencing; controls; NA | NJ (Monmouth) | Ex. vegetation |
|  | *Bm* | N: 7/250 |  |  |  |
|  | *Bl* | N: 0/250 |  |  |  |
| Walk[76]; 2007 | *Bb* | A: 266/509 | qPCR, sequencing; no controls; NA | NH (Coos, Grafton, Sullivan, Cheshire, Hillsborough, Rockingham, Merrimack, Belknap, Carroll) | Flagging |
|  | *Ap* | A: 1/509 |  |  |  |
|  | *Bam* | A: 15/509 |  |  |  |
|  | *Bb + Ap* | A: 9/509 |  |  |  |
|  | *Bb + Bam* | A: 31/509 |  |  |  |
|  |  |  |  |  |  |
|  |  |  |  |  |  |
|  |  |  |  |  |  |
| Williamson[77]; 2004-08 | *Ehrlichia* spp. | N/A: 0/76 | Nested PCR, sequencing; controls; NG | TX (unknown) | Ex. humans |
|  | *Bb* | N/A: 1/76 |  |  |  |
|  | *R. peacockii* | N/A: 1/76 |  |  |  |
|  | *Candidatus* Rickettsia cooleyi | N/A: 35/76 |  |  |  |
| Yabsley[78]; 2003-06 | *R. cooleyi*-like | A: 7/52 | PCR, sequencing; no controls; NG | FL (Bay, Franklin, Gulf, Jefferson, Liberty, Madison, Okaloosa, Wakulla) | Ex. black beers |
|  | *Rickettsia* sp. TR39 | A: 18/52 |  |  |  |
|  | *Rickettsia* sp. Is-I | A: 1/52 |  |  |  |
|  | *Ap* | A: 0/52 |  |  |  |
|  | *E. ewingii* | A: 0/52 |  |  |  |
|  | *F. tularensis* | A: 0/52 |  |  |  |
|  | Unidentified *Borrelia* spp. | A: 0/52 |  |  |  |
|  | *Ec* | A: 0/52 |  |  |  |
|  | *Panola Mountain Ehrlichia* sp. | A: 0/52 |  |  |  |
|  | *R. cooleyi*-like | A: 2/13 |  | GA (Charlton, Clinch, Echols, Ware) |  |
|  | *Rickettsia* sp. TR39 | A: 1/13 |  |  |  |
|  | *Rickettsia* sp. Is-I | A: 0/13 |  |  |  |
|  | *Ap* | A: 0/13 |  |  |  |
|  | *E. ewingii* | A: 0/13 |  |  |  |
|  | *F. tularensis* | A: 0/13 |  |  |  |
|  | *Borrelia* spp. | A: 0/13 |  |  |  |
|  | *Ec* | A: 0/13 |  |  |  |
|  | Panola Mountain *Ehrlichia* sp. | A: 0/13 |  |  |  |

*dates indicate year(s) studies were performed

**tick stage: U, unknown stage; L, larva; N, nymph; A, adult

^†^ESI-MS, electrospray ionisation mass spectrometry; PCR, polymerase chain reaction; RT-PCR, reverse transcription-PCR; qPCR, real-time or quantitative PCR; MASTAQ-PCR, Middleware Architecture for Sensor Applications with Statistical Quality-PCR; MLST, multilocus sequence typing; RFLP, restriction fragment length polymorphism; TTGE, temperature gradient gel electrophoresis; TEM, transmission electron microscopy; SNP, single nucleotide polymorphism

^‡^NG, no GenBank submission made; NA, not applicable

^‡‡^**Canada:** AB, Alberta; MB, Manitoba; NB, New Brunswick; NL, Newfoundland; NS, Nova Scotia; ON, Ontario; PE, Prince Edward Island; SK, Saskatchewan; QC, Quebec. **US:** AR, Arkansas; CT, Connecticut; DE, Delaware; FL, Florida; GA, Georgia; IL, Illinois; IN, Indiana; IA, Iowa; KY, Kentucky; LA, Louisiana; ME, Maine; MD, Maryland; MA, Massachusetts; MI, Michigan; MN, Minnesota; MS, Mississippi; NH, New Hampshire; NJ, New Jersey; NY, New York; NC, North Carolina; ND, North Dakota; PA, Pennsylvania; RI, Rhode Island; SC, South Carolina; TN, Tennessee; TX, Texas; VT, Vermont; VA, Virginia; WI, Wisconsin

**References**

1. Adelson ME, Rao RV, Tilton RC, Cabets K, Eskow E, Fein L, et al. Prevalence of *Borrelia burgdorferi*, *Bartonella* spp., *Babesia microti*, and *Anaplasma phagocytophila* in *Ixodes scapularis* ticks collected in Northern New Jersey. J Clin Microbiol. 2004;42:2799-801.
2. Aliota MT, Dupuis AP, Wilczek MP, Peters RJ, Ostfeld RS, Kramer LD. The prevalence of zoonotic tick-borne pathogens in *Ixodes scapularis* collected in the Hudson Valley, New York State*.* Vector Borne Zoonotic Dis. 2014;14:245-50.
3. Anderson JF, Armstrong PM. Prevalence and genetic characterization of Powassan virus strains infecting *Ixodes scapularis* in Connecticut*.* Am J Trop Med Hyg. 2012;87:754-9.
4. Barbour AG, Bunikis J, Travinsky B, Hoen AG, Diuk-Wasser MA, Fish D, et al. Niche partitioning of *Borrelia burgdorferi* and *Borrelia miyamotoi* in the same tick vector and mammalian reservoir species*.* Am J Trop Med Hyg. 2009;81:1120-31.
5. Benson MJ, Gawronski JD, Eveleigh DE, Benson DR. Intracellular symbionts and other bacteria associated with deer ticks (*Ixodes scapularis*) from Nantucket and Wellfleet, Cape Cod, Massachusetts*.* Appl Environ Microbiol. 2004;70:616-20.
6. Bouchard C, Leighton PA, Beauchamp G, Nguon S, Trudel L, Milord F, et al. Harvested white-tailed deer as sentinel hosts for early establishing *Ixodes scapularis* populations and risk from vector-borne zoonoses in southeastern Canada*.* J Med Entomol. 2013;50:384-93.
7. Brackney DE, Nofchissey RA, Fitzpatrick KA, Brown IK, Ebel GD. Stable prevalence of Powassan virus in *Ixodes scapularis* in a northern Wisconsin focus*.* Am J Trop Med Hyg. 2008;79:971-3.
8. Cherepko J, Berry GJ, Keeler SP, Huffman JE. Prevalence of *Borrelia burgdorferi*, *Bartonella* spp., *Bartonella henselae*, *Babesia microti* and *Anaplasma phagocytophila* in *Ixodes scapularis* ticks collected in Monroe County, Pennsylvania, show a risk for co- and tri-infections*.* J Pa Acad Sci. 2010;84:74-8.
9. Cohen SB, Yabsley MJ, Freye JD, Dunlap BG, Rowland ME, Huang J, et al. Prevalence of *Ehrlichia chaffeensis* and *Ehrlichia ewingii* in ticks from Tennessee*.* Vector Borne Zoonotic Dis. 2010;10:435-40.
10. Courtney JW, Dryden RL, Montgomery J, Schneider BS, Smith G, Massung RF. Molecular characterization of *Anaplasma phagocytophilum* and *Borrelia burgdorferi* in *Ixodes scapularis* ticks from Pennsylvania*.* J Clin Microbiol. 2003;41:1569-73.
11. Crowder CD, Carolan HE, Rounds MA, Honig V, Mothes B, Haag H, et al. Prevalence of *Borrelia miyamotoi* in *Ixodes* ticks in Europe and the United States*.* Emerg Infect Dis. 2014;20:1678-82.
12. Curran KL, Kidd JB, Vassallo J, Van Meter VL. *Borrelia burgdorferi* and the causative agent of human granulocytic ehrlichiosis in deer ticks, Delaware*.* Emerg Infect Dis. 2000;6:408-11.
13. Dibernardo A, Cote T, Ogden NH, Lindsay LR. The prevalence of *Borrelia miyamotoi* infection, and co-infections with other *Borrelia* spp. in *Ixodes scapularis* ticks collected in Canada*.* Parasit Vectors. 2014;doi: 10.1186/1756-3305-7-183.
14. Drebot MA, Lindsay R, Barker IK, Artsob H. Characterization of a human granulocytic ehrlichiosis-like agent from *Ixodes scapularis*, Ontario, Canada*.* Emerg Infect Dis. 2001;7:479-80.
15. Diuk-Wasser MA, Liu Y, Steeves TK, Folsom-O'Keefe C, Dardick KR, Lepore T, et al. Monitoring human babesiosis emergence through vector surveillance New England, USA*.* Emerg Infect Dis. 2014;20:225-31.
16. Dupuis AP, Peters RJ, Prusinski MA, Falco RC, Ostfeld RS, Kramer LD. Isolation of deer tick virus (Powassan virus, lineage II) from *Ixodes scapularis* and detection of antibody in vertebrate hosts sampled in the Hudson Valley, New York State*.* Parasit Vectors. 2013;doi: 10.1186/1756-3305-6-185.
17. Ebel GD, Campbell EN, Goethert HK, Spielman A, Telford SR. Enzootic transmission of deer tick virus in New England and Wisconsin sites*.* Am J Trop Med Hyg. 2000;63:36-42.
18. Eskow E, Rao RV, Mordechai E. Concurrent infection of the central nervous system by *Borrelia burgdorferi* and *Bartonella henselae*: evidence for a novel tick-borne disease complex*.* Arch Neurol. 2001;58:1357-63.
19. Fang QQ, Mixson TR, Hughes M, Dunham B, Sapp J. Prevalence of the agent of human granulocytic ehrlichiosis in *Ixodes scapularis* (Acari: Ixodidae) in the coastal southeastern United States*.* J Med Entomol. 2002;39:251-5.
20. Fritzen C, Mosites E, Applegate RD, Telford SR, Huang J, Yabsley MJ, et al. Environmental investigation following the first human case of babesiosis in Tennessee*.* J Parasitol. 2014;100:106-9.
21. Goltz L, Varela-Stokes A, Goddard J. Survey of adult *Ixodes scapularis* Say for disease agents in Mississippi*.* J Vector Ecol. 2013;38:401-3.
22. Grant-Klein RJ, Baldwin CD, Turell MJ, Rossi CA, Li F, Lovari R, et al. Rapid identification of vector-borne flaviviruses by mass spectrometry*.* Mol Cell Probes. 2010;24:219-28.
23. Hamer SA, Hickling GJ, Keith R, Sidge JL, Walker ED, Tsao JI. Associations of passerine birds, rabbits, and ticks with *Borrelia miyamotoi* and *Borrelia andersonii* in Michigan, U.S.A*. Parasit Vectors*. 2012;5:doi: 10.1186/1756-3305-5-231.
24. Hamer SA, Tsao JI, Walker ED, Mansfield LS, Foster ES, Hickling GJ. Use of tick surveys and serosurveys to evaluate pet dogs as a sentinel species for emerging Lyme disease*.* Am J Vet Res. 2009;70:49-56.
25. Hamer SA, Roy PL, Hickling GJ, Walker ED, Foster ES, Barber CC, et al. Zoonotic pathogens in *Ixodes scapularis*, Michigan*.* Emerg Infect Dis. 2007;13:1131-3.
26. Hamer SA, Hickling GJ, Walker ED, Tsao JI. Increased diversity of zoonotic pathogens and *Borrelia burgdorferi* strains in established versus incipient *Ixodes scapularis* populations across the Midwestern United States*.* Infect Genet Evol. 2014;27:531-42.
27. Hamer SA, Goldberg TL, Kitron UD, Brawn JD, Anderson TK, Loss SR, et al. Wild birds and urban ecology of ticks and tick-borne pathogens, Chicago, Illinois, USA, 2005-2010*.* Emerg Infect Dis. 2012;18:1589-95.
28. Hamer SA, Lehrer E, Magle SB. Wild birds as sentinels for multiple zoonotic pathogens along an urban to rural gradient in greater Chicago, Illinois*.* Zoonoses Public Health. 2012;59:355-64.
29. Han GS, Stromdahl EY, Wong D, Weltman AC. Exposure to *Borrelia burgdorferi* and other tick-borne pathogens in Gettysburg National Military Park, South-Central Pennsylvania, 2009*.* Vector Borne Zoonotic Dis. 2014;14:227-33.
30. Herrin BH, Zajac AM, Little SE. Confirmation of *Borrelia burgdorferi* sensu stricto and *Anaplasma phagocytophilum* in *Ixodes scapularis*, Southwestern Virginia*.* Vector Borne Zoonotic Dis. 2014;14:821-3.
31. Hersh MH, Tibbetts M, Strauss M, Ostfeld RS, Keesing F. Reservoir competence of wildlife host species for *Babesia microti.* Emerg Infect Dis. 2012;18:1951-7.
32. Hersh MH, Ostfeld RS, McHenry DJ, Tibbetts M, Brunner JL, Killilea ME, et al. Co-infection of blacklegged ticks with *Babesia microti* and *Borrelia burgdorferi* is higher than expected and acquired from small mammal hosts*.* PLoS One. 2014;9:e99348.
33. Hoen AG, Rollend LG, Papero MA, Carroll JF, Daniels TJ, Mather TN, et al. Effects of tick control by acaricide self-treatment of white-tailed deer on host-seeking tick infection prevalence and entomologic risk for *Ixodes scapularis*-borne pathogens*.* Vector Borne Zoonotic Dis. 2009;9:431-8.
34. Holman MS, Caporale DA, Goldberg J, Lacombe E, Lubelczyk C, Rand PW, et al. *Anaplasma phagocytophilum*, *Babesia microti*, and *Borrelia burgdorferi* in *Ixodes scapularis*, southern coastal Maine*.* Emerg Infect Dis. 2004;10:744-6.
35. Keesing F, McHenry DJ, Hersh M, Tibbetts M, Brunner JL, Killilea M, et al. Prevalence of human-active and variant 1 strains of the tick-borne pathogen *Anaplasma phagocytophilum* in hosts and forests of eastern North America*.* Am J Trop Med Hyg. 2014;91:302-9.
36. Kogut SJ, Thill CD, Prusinski MA, Lee JH, Backerson PB, Coleman JL, et al. *Babesia microti*, upstate New York*.* Emerg Infect Dis. 2005;11:476-8.
37. Krakowetz CN, Dibernardo A, Lindsay LR, Chilton NB. Two *Anaplasma phagocytophilum* strains in *Ixodes scapularis* ticks, Canada*.* Emerg Infect Dis. 2014;20:2064-7.
38. Kurtti TJ, Felsheim RF, Burkhardt NY, Oliver JD, Heu CC, Munderloh UG. *Rickettsia buchneri* sp. nov., a rickettsial endosymbiont of the blacklegged tick *Ixodes scapularis.* Int J Syst Evol Microbiol. 2015;65:965-70.
39. Layfield D, Guilfoile P. The prevalence of *Borrelia burgdorfieri* (Spirochaetales: spirochaetaceae) and the agent of human granulocytic ehrlichiosis (Rickettsiaceae: Ehrlichieae) in *Ixodes scapularis* (Acari: Ixodidae) collected during 1998 and 1999 from Minnesota*.* J Med Entomol. 2002;39:218-20.
40. Lee X, Coyle DR, Johnson DK, Murphy MW, McGeehin MA, Murphy RJ, et al. Prevalence of *Borrelia burgdorferi* and *Anaplasma phagocytophilum* in *Ixodes scapularis* (Acari: Ixodidae) nymphs collected in managed red pine forests in Wisconsin*.* J Med Entomol. 2014;51:694-701.
41. Leydet BF, Liang FT. Detection of Lyme *Borrelia* in questing *Ixodes scapularis* (Acari: Ixodidae) and small mammals in Louisiana*.* J Med Entomol. 2014;51:278-82.
42. Lovrich SD, Jobe DA, Kowalski TJ, Policepatil SM, Callister SM. Expansion of the Midwestern focus for human granulocytic anaplasmosis into the region surrounding La Crosse, Wisconsin*.* J Clin Microbiol. 2011;49:3855-9.
43. Margos G, Hojgaard A, Lane RS, Cornet M, Fingerle V, Rudenko N, et al. Multilocus sequence analysis of *Borrelia bissettii* strains from North America reveals a new *Borrelia* species, *Borrelia kurtenbachii.* Ticks Tick Borne Dis. 2010;1:151-8.
44. Massung RF, Mauel MJ, Owens JH, Allan N, Courtney JW, Stafford KC, et al. Genetic variants of *Ehrlichia phagocytophila*, Rhode Island and Connecticut*.* Emerg Infect Dis. 2002;8:467-72.
45. Massung RF, Mather TN, Priestley RA, Levin ML. Transmission efficiency of the AP-variant 1 strain of *Anaplasma phagocytophila.* Ann N Y Acad Sci. 2003;990:75-9.
46. Mays SE, Hendricks BM, Paulsen DJ, Houston AE, Trout Fryxell RT. Prevalence of five tick-borne bacterial genera in adult *Ixodes scapularis* removed from white-tailed deer in western Tennessee*.* Parasit Vectors. 2014;7:doi: 10.1186/s13071-014-0473-y.
47. McCall JW, Baker CF, Mather TN, Chester ST, McCall SD, Irwin JP, et al. The ability of a topical novel combination of fipronil, amitraz and (S)-methoprene to protect dogs from *Borrelia burgdorferi* and *Anaplasma phagocytophilum* infections transmitted by *Ixodes scapularis.* Vet Parasitol. 2011;179:335-42.
48. Michalski M, Rosenfield C, Erickson M, Selle R, Bates K, Essar D, et al. *Anaplasma phagocytophilum* in central and western Wisconsin: a molecular survey*.* Parasitol Res. 2006;99:694-9.
49. Moreno CX, Moy F, Daniels TJ, Godfrey HP, Cabello FC. Molecular analysis of microbial communities identified in different developmental stages of *Ixodes scapularis* ticks from Westchester and Dutchess Counties, New York*.* Environ Microbiol. 2006;8:761-72.
50. Moncayo AC, Cohen SB, Fritzen CM, Huang E, Yabsley MJ, Freye JD, et al. Absence of *Rickettsia rickettsii* and occurrence of other spotted fever group rickettsiae in ticks from Tennessee*.* Am J Trop Med Hyg. 2010;83:653-7.
51. Nelder MP, Russell C, Lindsay LR, Dhar B, Patel SN, Johnson S, et al. Population-based passive tick surveillance and detection of expanding foci of blacklegged ticks *Ixodes scapularis* and the Lyme disease agent *Borrelia burgdorferi* in Ontario, Canada*.* PLoS One. 2014;e105358.
52. Ogden NH, Lindsay LR, Hanincova K, Barker IK, Bigras-Poulin M, Charron DF, et al. Role of migratory birds in introduction and range expansion of *Ixodes scapularis* ticks and of *Borrelia burgdorferi* and *Anaplasma phagocytophilum* in Canada*.* Appl Environ Microbiol. 2008;74:1780-90.
53. Ogden NH, Margos G, Aanensen DM, Drebot MA, Feil EJ, Hanincova K, et al. Investigation of genotypes of *Borrelia burgdorferi* in *Ixodes scapularis* ticks collected during surveillance in Canada*.* Appl Environ Microbiol. 2011;77:3244-54.
54. Pritt BS, Sloan LM, Johnson DK, Munderloh UG, Paskewitz SM, McElroy KM, et al. Emergence of a new pathogenic *Ehrlichia* species, Wisconsin and Minnesota, 2009*.* N Engl J Med. 2011;365:422-9.
55. Prusinski MA, Kokas JE, Hukey KT, Kogut SJ, Lee J, Backenson PB. Prevalence of *Borrelia burgdorferi* (Spirochaetales: Spirochaetaceae), *Anaplasma phagocytophilum* (Rickettsiales: Anaplasmataceae), and *Babesia microti* (Piroplasmida: Babesiidae) in *Ixodes scapularis* (Acari: Ixodidae) collected from recreational lands in the Hudson Valley Region, New York State*.* J Med Entomol. 2014;51:226-36.
56. Roellig DM, Fang QQ. Detection of *Anaplasma phagocytophilum* in ixodid ticks from equine-inhabited sites in the Southeastern United States*.* Vector Borne Zoonotic Dis. 2012;12:330-2.
57. Rollend L, Bent SJ, Krause PJ, Usmani-Brown S, Steeves TK, States SL, et al. Quantitative PCR for detection of *Babesia microti* in *Ixodes scapularis* ticks and in human blood*.* Vector Borne Zoonotic Dis. 2013;13:784-90.
58. Russart NM, Dougherty MW, Vaughan JA. Survey of ticks (Acari: Ixodidae) and tick-borne pathogens in North Dakota*.* J Med Entomol. 2014;51:1087-90.
59. Schulze TL, Jordan RA, Healy SP, Roegner VE, Meddis M, Jahn MB, et al. Relative abundance and prevalence of selected *Borrelia* infections in *Ixodes scapularis* and *Amblyomma americanum* (Acari: Ixodidae) from publicly owned lands in Monmouth County, New Jersey*.* J Med Entomol. 2006;43:1269-75.
60. Schulze TL, Jordan RA, Schulze CJ, Mixson T, Papero M. Relative encounter frequencies and prevalence of selected *Borrelia*, *Ehrlichia*, and *Anaplasma* infections in *Amblyomma americanum* and *Ixodes scapularis* (Acari: Ixodidae) ticks from central New Jersey*.* J Med Entomol. 2005;42:450-6.
61. Schulze TL, Jordan RA, Healy SP, Roegner VE. Detection of *Babesia microti* and *Borrelia burgdorferi* in host-seeking *Ixodes scapularis* (Acari: Ixodidae) in Monmouth County, New Jersey*.* J Med Entomol. 2013;50:379-83.
62. Scoles GA, Papero M, Beati L, Fish D. A relapsing fever group spirochete transmitted by *Ixodes scapularis* ticks*.* Vector Borne Zoonotic Dis. 2001;1:21-34.
63. Shukla SK, Vandermause MF, Belongia EA, Reed KD, Paskewitz SM, Kazmierczak J. Importance of primer specificity for PCR detection of *Anaplasma phagocytophila* among *Ixodes scapularis* ticks from Wisconsin*.* J Clin Microbiol. 2003;41:4006.
64. Smith RP,Jr, Elias SP, Borelli TJ, Missaghi B, York BJ, Kessler RA, et al. Human babesiosis, Maine, USA, 1995-2011*.* Emerg Infect Dis. 2014;20:1727-30.
65. Smith MP, Ponnusamy L, Jiang J, Ayyash LA, Richards AL, Apperson CS. Bacterial pathogens in ixodid ticks from a Piedmont County in North Carolina: prevalence of rickettsial organisms*.* Vector Borne Zoonotic Dis. 2010;10:939-52.
66. Steiner FE, Pinger RR, Vann CN, Abley MJ, Sullivan B, Grindle N, et al. Detection of *Anaplasma phagocytophilum* and *Babesia odocoilei* DNA in *Ixodes scapularis* (Acari: Ixodidae) collected in Indiana*.* J Med Entomol. 2006;43:437-42.
67. Steiner FE, Pinger RR, Vann CN, Grindle N, Civitello D, Clay K, et al. Infection and co-infection rates of *Anaplasma phagocytophilum* variants, *Babesia* spp., *Borrelia burgdorferi*, and the rickettsial endosymbiont in *Ixodes scapularis* (Acari: Ixodidae) from sites in Indiana, Maine, Pennsylvania, and Wisconsin*.* J Med Entomol. 2008;45:289-97.
68. Swanson KI, Norris DE. Co-circulating microorganisms in questing *Ixodes scapularis* nymphs in Maryland*.* J Vector Ecol. 2007;32:243-51.
69. Taft SC, Miller MK, Wright SM. Distribution of borreliae among ticks collected from eastern states*.* Vector Borne Zoonotic Dis. 2005;5:383-9.
70. Telford ISR, Goethert HK, Cunningham JA. Prevalence of *Ehrlichia muris* in Wisconsin deer ticks collected during the mid 1990s*.* Open Microbiol J. 2011;5:18-20.
71. Tokarz R, Kapoor V, Samuel JE, Bouyer DH, Briese T, Lipkin WI. Detection of tick-borne pathogens by MassTag polymerase chain reaction*.* Vector Borne Zoonotic Dis. 2009;9:147-52.
72. Tokarz R, Jain K, Bennett A, Briese T, Lipkin WI. Assessment of polymicrobial infections in ticks in New York state*.* Vector Borne Zoonotic Dis. 2010;10:217-21.
73. Tokarz R, Williams SH, Sameroff S, Sanchez Leon M, Jain K, Lipkin WI. Virome analysis of *Amblyomma americanum*, *Dermacentor variabilis*, and *Ixodes scapularis* ticks reveals novel highly divergent vertebrate and invertebrate viruses*.* J Virol. 2014;88:11480-92.
74. Fryxell RT, Steelman CD, Szalanski AL, Kvamme KL, Billingsley PM, Williamson PC. Survey of Borreliae in ticks, canines, and white-tailed deer from Arkansas, U.S.A*.* Parasit Vectors. 2012; 5:doi: 10.1186/1756-3305-5-139.
75. Ullmann AJ, Gabitzsch ES, Schulze TL, Zeidner NS, Piesman J. Three multiplex assays for detection of *Borrelia burgdorferi* sensu lato and *Borrelia miyamotoi* sensu lato in field-collected *Ixodes* nymphs in North America*.* J Med Entomol. 2005;42:1057-62.
76. Walk ST, Xu G, Stull JW, Rich SM. Correlation between tick density and pathogen endemicity, New Hampshire*.* Emerg Infect Dis. 2009;15:585-7.
77. Williamson PC, Billingsley PM, Teltow GJ, Seals JP, Turnbough MA, Atkinson SF. *Borrelia*, *Ehrlichia*, and *Rickettsia* spp. in ticks removed from persons, Texas, USA*.* Emerg Infect Dis. 2010;16:441-6.
78. Yabsley MJ, Nims TN, Savage MY, Durden LA. Ticks and tick-borne pathogens and putative symbionts of black bears (*Ursus americanus floridanus*) from Georgia and Florida*.* J Parasitol. 2009;95:1125-8.
