# Supplementary material for: Human pathogens associated with the blacklegged tick Ixodes scapularis: a systematic review
Source: Parasit Vectors. 2016 May 5;9:265. doi: 10.1186/s13071-016-1529-y (PMC4857413; doi:10.1186/s13071-016-1529-y)
Supplement: Additional file 3: — Critical appraisal of 78 studies in review. (DOCX 61 kb) [file 13071_2016_1529_MOESM3_ESM.docx]

**Additional file 3.** Critical appraisal of 78 reviewed studies

| Study identification | | Assessment of relevancy | Assessment of reliability |  | Assessment of validity |  |  |  | Assessment of applicability |
| --- | --- | --- | --- | --- | --- | --- | --- | --- | --- |
| Year | **First author, reference** | **a) Does the study address a topic(s) relevant to the issue under investigation?** | **a) Is the study presented clearly?** | **b) Are the research methodology and results clearly described?** | **a) Is the study methodology appropriate for the scope of research?** | **b) Is the research methodology free from bias?** | **c) Are the authors' conclusions explicit and transparent?** | **d) Can I be confident about the findings?** | **Can the results be applied within the scope of public health?** |
|  |  | **[1]** Was the justification for the study clearly stated?  **[2]** Do the results of the study apply to the issue under consideration?  **[3]** How similar or different is the study population or setting to yours? Is a difference likely to matter for the issue at hand? | **[1]** Is the rationale for study clearly stated, and does the study focus on a clearly defined issue?  **[2]** Can the study be reproduced with the information provided? | **[1]** Are tick collection methods defined?  **[2]** Are host species reported?  **[3]** Are positive and negative controls explicitly and clearly described?  **[4]** Is the tick stage identified?  **[5]** Are collection locales clearly identified?  [6] Is GenBank submission included where applicable | **[1]** Is the research question congruent with the study design? | **[1]** Were there major sources of bias with respect to: study design?  **[2]** Are the results consistent within the study? **[3]** Can chance findings be ruled out? | **[1]** Are the results conclusive?  **[2]** Are the authors' conclusions clearly derived from the results  **[3]** Are potential discrepancies discussed?  **[4]** Are limitations of work described? | **[1]** Are there any major methodological flaws that limit the validity of the findings? | **[1]** Can the study results be interpreted and analyzed within the context of public health? |
| 2004 | **Adelson [1]** | Yes | Yes | Stage not described | Yes | Yes | Yes | No | Yes |
| 2014 | **Aliota [2]** | Yes | Yes | Yes | Yes | Yes | Yes | No | Yes |
| 2012 | **Anderson [3]** | Yes | Yes | No negative controls | Yes | Yes | Yes | No | Yes |
| 2009 | **Barbour [4]** | Yes | Yes | No positive controls | Yes | Yes | Yes | No | Yes |
| 2004 | **Benson [5]** | Yes | Yes | No positive controls | Yes | Yes | Yes | No | Yes |
| 2013 | **Bouchard [6]** | Yes | Yes | No controls | Yes | Yes | Yes | No | Yes |
| 2008 | **Brackney [7]** | Yes | Yes | No controls | Yes | Yes | Yes | No | Yes |
| 2010 | **Cherepko [8]** | Yes | Yes | Yes | Yes | Yes | Yes | No | Yes |
| 2010 | **Cohen [9]** | Yes | Yes | No negative controls | Yes | Small sample size | Yes | Yes/No | Yes |
| 2003 | **Courtney [10]** | Yes | Yes | No controls | Yes | Yes | Yes | No | Yes |
| 2014 | **Crowder [11]** | Yes | Yes | Yes | Yes | Yes | Yes | No | Yes |
| 2000 | **Curran [12]** | Yes | Yes | Yes | Yes | Yes | Yes | No | Yes |
| 2014 | **Dibernardo [13]** | Yes | Yes | Yes | Yes | Yes | Yes | No | Yes |
| 2001 | **Drebot [14]** | Yes | Yes | No controls | Yes | Yes | Yes | No | Yes |
| 2014 | **Diuk-Wasser [15]** | Yes | Yes | No controls | Yes | Yes | Yes | No | Yes |
| 2013 | **Dupuis [16]** | Yes | Yes | No controls | Yes | Yes | Yes | No | Yes |
| 2000 | **Ebel [17]** | Yes | Yes | No negative controls | Yes | Yes | Yes | No | Yes |
| 2001 | **Eskow [18]** | Yes | Yes | Study location not exact | Yes | Small sample size | Yes | No | Yes |
| 2002 | **Fang [19]** | Yes | Yes | Yes | Yes | Yes | Yes | No | Yes |
| 2014 | **Fritzen [20]** | Yes | Yes | Yes | Yes | Yes | Yes | No | Yes |
| 2013 | **Goltz [21]** | Yes | Yes | No negative controls | Yes | Yes | Yes | No | Yes |
| 2010 | **Grant-Klein [22]** | Yes | Yes | No negative controls; No GenBank | Yes | Yes | Yes | Yes/No | Yes |
| 2012 | **Hamer [23]** | Yes | Yes | No negative controls | Yes | Yes | Yes | No | Yes |
| 2009 | **Hamer [24]** | Yes | Yes | No negative controls | Yes | Yes | Yes | No | Yes |
| 2007 | **Hamer [25]** | Yes | Yes | No controls | Yes | Yes | Yes | No | Yes |
| 2014 | **Hamer [26]** | Yes | Yes | Yes | Yes | Yes | Yes | No | Yes |
| 2012 | **Hamer [27]** | Yes | Yes | No controls | Yes | Yes | Yes | No | Yes |
| 2012 | **Hamer [28]** | Yes | Yes | Yes | Yes | Small sample size | Yes | No | Yes |
| 2014 | **Han [29]** | Yes | Yes | No controls | Yes | Yes | Yes | No | Yes |
| 2014 | **Herrin [30]** | Yes | Yes | No controls | Yes | Yes | Yes | No | Yes |
| 2014 | **Hersh [31]** | Yes | Yes | No controls | Yes | Yes | Yes | No | Yes |
| 2012 | **Hersh [32]** | Yes | Yes | Yes | Yes | Small sample size | Yes | No | Yes |
| 2009 | **Hoen [33]** | Yes | Yes | Yes | Yes | Yes | Yes | No | Yes |
| 2004 | **Holman [34]** | Yes | Yes | Yes | Yes | Yes | Yes | No | Yes |
| 2014 | **Keesing [35]** | Yes | Yes | Unknown stage in one | Yes | Yes | Yes | No | Yes |
| 2005 | **Kogut [36]** | Yes | Yes | No positive controls | Yes | Yes | Yes | No | Yes |
| 2014 | **Krakowetz [37]** | Yes | Yes | No controls; stages of tick not reported | Yes | Yes | Yes | No | Yes |
| 2015 | **Kurtti [38]** | Yes | Yes | No controls | Yes | Yes | Yes | No | Yes |
| 2002 | **Layfield [39]** | Yes | Yes | Unknown tick stages | Yes | Yes | Yes | No | Yes |
| 2014 | **Lee [40]** | Yes | More detail needed to reproduce | Unknown stages; unknown collection methods | Yes | Yes | Yes | Yes/No | Yes |
| 2013 | **Leydet [41]** | Yes | Yes | No controls; No GenBank | Yes | Small sample size | Yes | Yes/No | Yes |
| 2011 | **Lovrich [42]** | Yes | Yes | Yes | Yes | Yes | Yes | No | Yes |
| 2014 | **Margos [43]** | Yes | Yes | No controls | Yes | Small sample size | Yes | Yes/No | Yes |
| 2002 | **Massung [44]** | Yes | More detail needed to reproduce | No controls; collection methods vague | Yes | Yes | Yes | Yes/No | Yes |
| 2003 | **Massung [45]** | Yes | More detail needed to reproduce | No controls; collection methods vague | Yes | Yes | Yes | Yes/No | Yes |
| 2014 | **Mays [46]** | Yes | Yes | Yes | Yes | Yes | Yes | No | Yes |
| 2011 | **McCall [47]** | Yes | Yes | No controls | Yes | Yes | Yes | No | Yes |
| 2006 | **Michalski [48]** | Yes | Yes | Unknown stage of ticks; no negative controls | Yes | Yes | Yes | No | Yes |
| 2006 | **Moreno [49]** | Yes | Yes | Yes | Yes | Yes | Yes | No | Yes |
| 2010 | **Moncayo [50]** | Yes | Yes | Yes | Yes | Small sample size | Yes | No | Yes |
| 2014 | **Nelder [51]** | Yes | Yes | No controls | Yes | Yes | Yes | No | Yes |
| 2008 | **Ogden [52]** | Yes | Yes | No controls | Yes | Yes | Yes | No | Yes |
| 2011 | **Ogden [53]** | Yes | Yes | No controls | Yes | Yes | Yes | No | Yes |
| 2011 | **Pritt [54]** | Yes | Yes | Yes | Yes | Yes | Yes | No | Yes |
| 2014 | **Prusinski [55]** | Yes | Yes | Yes | Yes | Yes | Yes | No | Yes |
| 2013 | **Roellig [56]** | Yes | Yes | No controls | Yes | Yes | Yes | No | Yes |
| 2013 | **Rollend [57]** | Yes | Yes | Yes | Yes | Yes | Yes | No | Yes |
| 2014 | **Russart [58]** | Yes | Yes | No controls | Yes | Yes | Yes | No | Yes |
| 2006 | **Schulze [59]** | Yes | Yes | No negative controls | Yes | Yes | Yes | No | Yes |
| 2005 | **Schulze [60]** | Yes | Yes | No GenBank | Yes | Yes | Yes | Yes/No | Yes |
| 2013 | **Schulze [61]** | Yes | Yes | No negative controls | Yes | Yes | Yes | No | Yes |
| 2001 | **Scoles [62]** | Yes | Yes | No GenBank | Yes | Yes | Yes | Yes/No | Yes |
| 2003 | **Shukla [63]** | Yes | Yes | No controls | Yes | Yes | Yes | No | Yes |
| 2014 | **Smith [64]** | Yes | Yes | No controls | Yes | Yes | Yes | No | Yes |
| 2010 | **Smith [65]** | Yes | Yes | No GenBank | Yes | Yes | Yes | Yes/No | Yes |
| 2006 | **Steiner [66]** | Yes | Yes | No controls | Yes | Yes | Yes | No | Yes |
| 2008 | **Steiner [67]** | Yes | Yes | Yes | Yes | Yes | Yes | No | Yes |
| 2007 | **Swanson [68]** | Yes | Yes | Yes | Yes | Yes | Yes | No | Yes |
| 2005 | **Taft [69]** | Yes | Yes | No positive controls | Yes | Yes | Yes | No | Yes |
| 2011 | **Telford [70]** | Yes | Yes | No controls | Yes | Yes | Yes | No | Yes |
| 2009 | **Tokarz [71]** | Yes | More detail needed to reproduce | Collection methods not described | Yes | Yes | Yes | Yes/No | Yes |
| 2010 | **Tokarz [72]** | Yes | Yes | No controls | Yes | Yes | Yes | No | Yes |
| 2014 | **Tokarz [73]** | Yes | Needs more detail to reproduce | No controls; collection methods not reported | Yes | Yes | Yes | Yes/No | Yes |
| 2012 | **Trout [74]** | Yes | Yes | Yes | Yes | Yes | Yes | No | Yes |
| 2005 | **Ullmann [75]** | Yes | Yes | Yes | Yes | Yes | Yes | No | Yes |
| 2009 | **Walk [76]** | Yes | Yes | No controls | Yes | Yes | Yes | No | Yes |
| 2010 | **Williamson [77]** | Yes | More detail needed to reproduce | No GenBank; unknown location in Texas | Yes | Yes | Yes | Yes/No | Yes |
| 2009 | **Yabsley [78]** | Yes | Yes | No controls, No GenBank | Yes | Yes | Yes | Yes/No | Yes |

**References**

1. Adelson ME, Rao RV, Tilton RC, Cabets K, Eskow E, Fein L, et al. Prevalence of *Borrelia burgdorferi*, *Bartonella* spp., *Babesia microti*, and *Anaplasma phagocytophila* in *Ixodes scapularis* ticks collected in Northern New Jersey. J Clin Microbiol. 2004;42:2799-801.
2. Aliota MT, Dupuis AP, Wilczek MP, Peters RJ, Ostfeld RS, Kramer LD. The prevalence of zoonotic tick-borne pathogens in *Ixodes scapularis* collected in the Hudson Valley, New York State*.* Vector Borne Zoonotic Dis. 2014;14:245-50.
3. Anderson JF, Armstrong PM. Prevalence and genetic characterization of Powassan virus strains infecting *Ixodes scapularis* in Connecticut*.* Am J Trop Med Hyg. 2012;87:754-9.
4. Barbour AG, Bunikis J, Travinsky B, Hoen AG, Diuk-Wasser MA, Fish D, et al. Niche partitioning of *Borrelia burgdorferi* and *Borrelia miyamotoi* in the same tick vector and mammalian reservoir species*.* Am J Trop Med Hyg. 2009;81:1120-31.
5. Benson MJ, Gawronski JD, Eveleigh DE, Benson DR. Intracellular symbionts and other bacteria associated with deer ticks (*Ixodes scapularis*) from Nantucket and Wellfleet, Cape Cod, Massachusetts*.* Appl Environ Microbiol. 2004;70:616-20.
6. Bouchard C, Leighton PA, Beauchamp G, Nguon S, Trudel L, Milord F, et al. Harvested white-tailed deer as sentinel hosts for early establishing *Ixodes scapularis* populations and risk from vector-borne zoonoses in southeastern Canada*.* J Med Entomol. 2013;50:384-93.
7. Brackney DE, Nofchissey RA, Fitzpatrick KA, Brown IK, Ebel GD. Stable prevalence of Powassan virus in *Ixodes scapularis* in a northern Wisconsin focus*.* Am J Trop Med Hyg. 2008;79:971-3.
8. Cherepko J, Berry GJ, Keeler SP, Huffman JE. Prevalence of *Borrelia burgdorferi*, *Bartonella* spp., *Bartonella henselae*, *Babesia microti* and *Anaplasma phagocytophila* in *Ixodes scapularis* ticks collected in Monroe County, Pennsylvania, show a risk for co- and tri-infections*.* J Pa Acad Sci. 2010;84:74-8.
9. Cohen SB, Yabsley MJ, Freye JD, Dunlap BG, Rowland ME, Huang J, et al. Prevalence of *Ehrlichia chaffeensis* and *Ehrlichia ewingii* in ticks from Tennessee*.* Vector Borne Zoonotic Dis. 2010;10:435-40.
10. Courtney JW, Dryden RL, Montgomery J, Schneider BS, Smith G, Massung RF. Molecular characterization of *Anaplasma phagocytophilum* and *Borrelia burgdorferi* in *Ixodes scapularis* ticks from Pennsylvania*.* J Clin Microbiol. 2003;41:1569-73.
11. Crowder CD, Carolan HE, Rounds MA, Honig V, Mothes B, Haag H, et al. Prevalence of *Borrelia miyamotoi* in *Ixodes* ticks in Europe and the United States*.* Emerg Infect Dis. 2014;20:1678-82.
12. Curran KL, Kidd JB, Vassallo J, Van Meter VL. *Borrelia burgdorferi* and the causative agent of human granulocytic ehrlichiosis in deer ticks, Delaware*.* Emerg Infect Dis. 2000;6:408-11.
13. Dibernardo A, Cote T, Ogden NH, Lindsay LR. The prevalence of *Borrelia miyamotoi* infection, and co-infections with other *Borrelia* spp. in *Ixodes scapularis* ticks collected in Canada*.* Parasit Vectors. 2014;doi: 10.1186/1756-3305-7-183.
14. Drebot MA, Lindsay R, Barker IK, Artsob H. Characterization of a human granulocytic ehrlichiosis-like agent from *Ixodes scapularis*, Ontario, Canada*.* Emerg Infect Dis. 2001;7:479-80.
15. Diuk-Wasser MA, Liu Y, Steeves TK, Folsom-O'Keefe C, Dardick KR, Lepore T, et al. Monitoring human babesiosis emergence through vector surveillance New England, USA*.* Emerg Infect Dis. 2014;20:225-31.
16. Dupuis AP, Peters RJ, Prusinski MA, Falco RC, Ostfeld RS, Kramer LD. Isolation of deer tick virus (Powassan virus, lineage II) from *Ixodes scapularis* and detection of antibody in vertebrate hosts sampled in the Hudson Valley, New York State*.* Parasit Vectors. 2013;doi: 10.1186/1756-3305-6-185.
17. Ebel GD, Campbell EN, Goethert HK, Spielman A, Telford SR. Enzootic transmission of deer tick virus in New England and Wisconsin sites*.* Am J Trop Med Hyg. 2000;63:36-42.
18. Eskow E, Rao RV, Mordechai E. Concurrent infection of the central nervous system by *Borrelia burgdorferi* and *Bartonella henselae*: evidence for a novel tick-borne disease complex*.* Arch Neurol. 2001;58:1357-63.
19. Fang QQ, Mixson TR, Hughes M, Dunham B, Sapp J. Prevalence of the agent of human granulocytic ehrlichiosis in *Ixodes scapularis* (Acari: Ixodidae) in the coastal southeastern United States*.* J Med Entomol. 2002;39:251-5.
20. Fritzen C, Mosites E, Applegate RD, Telford SR, Huang J, Yabsley MJ, et al. Environmental investigation following the first human case of babesiosis in Tennessee*.* J Parasitol. 2014;100:106-9.
21. Goltz L, Varela-Stokes A, Goddard J. Survey of adult *Ixodes scapularis* Say for disease agents in Mississippi*.* J Vector Ecol. 2013;38:401-3.
22. Grant-Klein RJ, Baldwin CD, Turell MJ, Rossi CA, Li F, Lovari R, et al. Rapid identification of vector-borne flaviviruses by mass spectrometry*.* Mol Cell Probes. 2010;24:219-28.
23. Hamer SA, Hickling GJ, Keith R, Sidge JL, Walker ED, Tsao JI. Associations of passerine birds, rabbits, and ticks with *Borrelia miyamotoi* and *Borrelia andersonii* in Michigan, U.S.A*. Parasit Vectors*. 2012;5:doi: 10.1186/1756-3305-5-231.
24. Hamer SA, Tsao JI, Walker ED, Mansfield LS, Foster ES, Hickling GJ. Use of tick surveys and serosurveys to evaluate pet dogs as a sentinel species for emerging Lyme disease*.* Am J Vet Res. 2009;70:49-56.
25. Hamer SA, Roy PL, Hickling GJ, Walker ED, Foster ES, Barber CC, et al. Zoonotic pathogens in *Ixodes scapularis*, Michigan*.* Emerg Infect Dis. 2007;13:1131-3.
26. Hamer SA, Hickling GJ, Walker ED, Tsao JI. Increased diversity of zoonotic pathogens and *Borrelia burgdorferi* strains in established versus incipient *Ixodes scapularis* populations across the Midwestern United States*.* Infect Genet Evol. 2014;27:531-42.
27. Hamer SA, Goldberg TL, Kitron UD, Brawn JD, Anderson TK, Loss SR, et al. Wild birds and urban ecology of ticks and tick-borne pathogens, Chicago, Illinois, USA, 2005-2010*.* Emerg Infect Dis. 2012;18:1589-95.
28. Hamer SA, Lehrer E, Magle SB. Wild birds as sentinels for multiple zoonotic pathogens along an urban to rural gradient in greater Chicago, Illinois*.* Zoonoses Public Health. 2012;59:355-64.
29. Han GS, Stromdahl EY, Wong D, Weltman AC. Exposure to *Borrelia burgdorferi* and other tick-borne pathogens in Gettysburg National Military Park, South-Central Pennsylvania, 2009*.* Vector Borne Zoonotic Dis. 2014;14:227-33.
30. Herrin BH, Zajac AM, Little SE. Confirmation of *Borrelia burgdorferi* sensu stricto and *Anaplasma phagocytophilum* in *Ixodes scapularis*, Southwestern Virginia*.* Vector Borne Zoonotic Dis. 2014;14:821-3.
31. Hersh MH, Tibbetts M, Strauss M, Ostfeld RS, Keesing F. Reservoir competence of wildlife host species for *Babesia microti.* Emerg Infect Dis. 2012;18:1951-7.
32. Hersh MH, Ostfeld RS, McHenry DJ, Tibbetts M, Brunner JL, Killilea ME, et al. Co-infection of blacklegged ticks with *Babesia microti* and *Borrelia burgdorferi* is higher than expected and acquired from small mammal hosts*.* PLoS One. 2014;9:e99348.
33. Hoen AG, Rollend LG, Papero MA, Carroll JF, Daniels TJ, Mather TN, et al. Effects of tick control by acaricide self-treatment of white-tailed deer on host-seeking tick infection prevalence and entomologic risk for *Ixodes scapularis*-borne pathogens*.* Vector Borne Zoonotic Dis. 2009;9:431-8.
34. Holman MS, Caporale DA, Goldberg J, Lacombe E, Lubelczyk C, Rand PW, et al. *Anaplasma phagocytophilum*, *Babesia microti*, and *Borrelia burgdorferi* in *Ixodes scapularis*, southern coastal Maine*.* Emerg Infect Dis. 2004;10:744-6.
35. Keesing F, McHenry DJ, Hersh M, Tibbetts M, Brunner JL, Killilea M, et al. Prevalence of human-active and variant 1 strains of the tick-borne pathogen *Anaplasma phagocytophilum* in hosts and forests of eastern North America*.* Am J Trop Med Hyg. 2014;91:302-9.
36. Kogut SJ, Thill CD, Prusinski MA, Lee JH, Backerson PB, Coleman JL, et al. *Babesia microti*, upstate New York*.* Emerg Infect Dis. 2005;11:476-8.
37. Krakowetz CN, Dibernardo A, Lindsay LR, Chilton NB. Two *Anaplasma phagocytophilum* strains in *Ixodes scapularis* ticks, Canada*.* Emerg Infect Dis. 2014;20:2064-7.
38. Kurtti TJ, Felsheim RF, Burkhardt NY, Oliver JD, Heu CC, Munderloh UG. *Rickettsia buchneri* sp. nov., a rickettsial endosymbiont of the blacklegged tick *Ixodes scapularis.* Int J Syst Evol Microbiol. 2015;65:965-70.
39. Layfield D, Guilfoile P. The prevalence of *Borrelia burgdorfieri* (Spirochaetales: spirochaetaceae) and the agent of human granulocytic ehrlichiosis (Rickettsiaceae: Ehrlichieae) in *Ixodes scapularis* (Acari: Ixodidae) collected during 1998 and 1999 from Minnesota*.* J Med Entomol. 2002;39:218-20.
40. Lee X, Coyle DR, Johnson DK, Murphy MW, McGeehin MA, Murphy RJ, et al. Prevalence of *Borrelia burgdorferi* and *Anaplasma phagocytophilum* in *Ixodes scapularis* (Acari: Ixodidae) nymphs collected in managed red pine forests in Wisconsin*.* J Med Entomol. 2014;51:694-701.
41. Leydet BF, Liang FT. Detection of Lyme *Borrelia* in questing *Ixodes scapularis* (Acari: Ixodidae) and small mammals in Louisiana*.* J Med Entomol. 2014;51:278-82.
42. Lovrich SD, Jobe DA, Kowalski TJ, Policepatil SM, Callister SM. Expansion of the Midwestern focus for human granulocytic anaplasmosis into the region surrounding La Crosse, Wisconsin*.* J Clin Microbiol. 2011;49:3855-9.
43. Margos G, Hojgaard A, Lane RS, Cornet M, Fingerle V, Rudenko N, et al. Multilocus sequence analysis of *Borrelia bissettii* strains from North America reveals a new *Borrelia* species, *Borrelia kurtenbachii.* Ticks Tick Borne Dis. 2010;1:151-8.
44. Massung RF, Mauel MJ, Owens JH, Allan N, Courtney JW, Stafford KC, et al. Genetic variants of *Ehrlichia phagocytophila*, Rhode Island and Connecticut*.* Emerg Infect Dis. 2002;8:467-72.
45. Massung RF, Mather TN, Priestley RA, Levin ML. Transmission efficiency of the AP-variant 1 strain of *Anaplasma phagocytophila.* Ann N Y Acad Sci. 2003;990:75-9.
46. Mays SE, Hendricks BM, Paulsen DJ, Houston AE, Trout Fryxell RT. Prevalence of five tick-borne bacterial genera in adult *Ixodes scapularis* removed from white-tailed deer in western Tennessee*.* Parasit Vectors. 2014;7:doi: 10.1186/s13071-014-0473-y.
47. McCall JW, Baker CF, Mather TN, Chester ST, McCall SD, Irwin JP, et al. The ability of a topical novel combination of fipronil, amitraz and (S)-methoprene to protect dogs from *Borrelia burgdorferi* and *Anaplasma phagocytophilum* infections transmitted by *Ixodes scapularis.* Vet Parasitol. 2011;179:335-42.
48. Michalski M, Rosenfield C, Erickson M, Selle R, Bates K, Essar D, et al. *Anaplasma phagocytophilum* in central and western Wisconsin: a molecular survey*.* Parasitol Res. 2006;99:694-9.
49. Moreno CX, Moy F, Daniels TJ, Godfrey HP, Cabello FC. Molecular analysis of microbial communities identified in different developmental stages of *Ixodes scapularis* ticks from Westchester and Dutchess Counties, New York*.* Environ Microbiol. 2006;8:761-72.
50. Moncayo AC, Cohen SB, Fritzen CM, Huang E, Yabsley MJ, Freye JD, et al. Absence of *Rickettsia rickettsii* and occurrence of other spotted fever group rickettsiae in ticks from Tennessee*.* Am J Trop Med Hyg. 2010;83:653-7.
51. Nelder MP, Russell C, Lindsay LR, Dhar B, Patel SN, Johnson S, et al. Population-based passive tick surveillance and detection of expanding foci of blacklegged ticks *Ixodes scapularis* and the Lyme disease agent *Borrelia burgdorferi* in Ontario, Canada*.* PLoS One. 2014;e105358.
52. Ogden NH, Lindsay LR, Hanincova K, Barker IK, Bigras-Poulin M, Charron DF, et al. Role of migratory birds in introduction and range expansion of *Ixodes scapularis* ticks and of *Borrelia burgdorferi* and *Anaplasma phagocytophilum* in Canada*.* Appl Environ Microbiol. 2008;74:1780-90.
53. Ogden NH, Margos G, Aanensen DM, Drebot MA, Feil EJ, Hanincova K, et al. Investigation of genotypes of *Borrelia burgdorferi* in *Ixodes scapularis* ticks collected during surveillance in Canada*.* Appl Environ Microbiol. 2011;77:3244-54.
54. Pritt BS, Sloan LM, Johnson DK, Munderloh UG, Paskewitz SM, McElroy KM, et al. Emergence of a new pathogenic *Ehrlichia* species, Wisconsin and Minnesota, 2009*.* N Engl J Med. 2011;365:422-9.
55. Prusinski MA, Kokas JE, Hukey KT, Kogut SJ, Lee J, Backenson PB. Prevalence of *Borrelia burgdorferi* (Spirochaetales: Spirochaetaceae), *Anaplasma phagocytophilum* (Rickettsiales: Anaplasmataceae), and *Babesia microti* (Piroplasmida: Babesiidae) in *Ixodes scapularis* (Acari: Ixodidae) collected from recreational lands in the Hudson Valley Region, New York State*.* J Med Entomol. 2014;51:226-36.
56. Roellig DM, Fang QQ. Detection of *Anaplasma phagocytophilum* in ixodid ticks from equine-inhabited sites in the Southeastern United States*.* Vector Borne Zoonotic Dis. 2012;12:330-2.
57. Rollend L, Bent SJ, Krause PJ, Usmani-Brown S, Steeves TK, States SL, et al. Quantitative PCR for detection of *Babesia microti* in *Ixodes scapularis* ticks and in human blood*.* Vector Borne Zoonotic Dis. 2013;13:784-90.
58. Russart NM, Dougherty MW, Vaughan JA. Survey of ticks (Acari: Ixodidae) and tick-borne pathogens in North Dakota*.* J Med Entomol. 2014;51:1087-90.
59. Schulze TL, Jordan RA, Healy SP, Roegner VE, Meddis M, Jahn MB, et al. Relative abundance and prevalence of selected *Borrelia* infections in *Ixodes scapularis* and *Amblyomma americanum* (Acari: Ixodidae) from publicly owned lands in Monmouth County, New Jersey*.* J Med Entomol. 2006;43:1269-75.
60. Schulze TL, Jordan RA, Schulze CJ, Mixson T, Papero M. Relative encounter frequencies and prevalence of selected *Borrelia*, *Ehrlichia*, and *Anaplasma* infections in *Amblyomma americanum* and *Ixodes scapularis* (Acari: Ixodidae) ticks from central New Jersey*.* J Med Entomol. 2005;42:450-6.
61. Schulze TL, Jordan RA, Healy SP, Roegner VE. Detection of *Babesia microti* and *Borrelia burgdorferi* in host-seeking *Ixodes scapularis* (Acari: Ixodidae) in Monmouth County, New Jersey*.* J Med Entomol. 2013;50:379-83.
62. Scoles GA, Papero M, Beati L, Fish D. A relapsing fever group spirochete transmitted by *Ixodes scapularis* ticks*.* Vector Borne Zoonotic Dis. 2001;1:21-34.
63. Shukla SK, Vandermause MF, Belongia EA, Reed KD, Paskewitz SM, Kazmierczak J. Importance of primer specificity for PCR detection of *Anaplasma phagocytophila* among *Ixodes scapularis* ticks from Wisconsin*.* J Clin Microbiol. 2003;41:4006.
64. Smith RP,Jr, Elias SP, Borelli TJ, Missaghi B, York BJ, Kessler RA, et al. Human babesiosis, Maine, USA, 1995-2011*.* Emerg Infect Dis. 2014;20:1727-30.
65. Smith MP, Ponnusamy L, Jiang J, Ayyash LA, Richards AL, Apperson CS. Bacterial pathogens in ixodid ticks from a Piedmont County in North Carolina: prevalence of rickettsial organisms*.* Vector Borne Zoonotic Dis. 2010;10:939-52.
66. Steiner FE, Pinger RR, Vann CN, Abley MJ, Sullivan B, Grindle N, et al. Detection of *Anaplasma phagocytophilum* and *Babesia odocoilei* DNA in *Ixodes scapularis* (Acari: Ixodidae) collected in Indiana*.* J Med Entomol. 2006;43:437-42.
67. Steiner FE, Pinger RR, Vann CN, Grindle N, Civitello D, Clay K, et al. Infection and co-infection rates of *Anaplasma phagocytophilum* variants, *Babesia* spp., *Borrelia burgdorferi*, and the rickettsial endosymbiont in *Ixodes scapularis* (Acari: Ixodidae) from sites in Indiana, Maine, Pennsylvania, and Wisconsin*.* J Med Entomol. 2008;45:289-97.
68. Swanson KI, Norris DE. Co-circulating microorganisms in questing *Ixodes scapularis* nymphs in Maryland*.* J Vector Ecol. 2007;32:243-51.
69. Taft SC, Miller MK, Wright SM. Distribution of borreliae among ticks collected from eastern states*.* Vector Borne Zoonotic Dis. 2005;5:383-9.
70. Telford ISR, Goethert HK, Cunningham JA. Prevalence of *Ehrlichia muris* in Wisconsin deer ticks collected during the mid 1990s*.* Open Microbiol J. 2011;5:18-20.
71. Tokarz R, Kapoor V, Samuel JE, Bouyer DH, Briese T, Lipkin WI. Detection of tick-borne pathogens by MassTag polymerase chain reaction*.* Vector Borne Zoonotic Dis. 2009;9:147-52.
72. Tokarz R, Jain K, Bennett A, Briese T, Lipkin WI. Assessment of polymicrobial infections in ticks in New York state*.* Vector Borne Zoonotic Dis. 2010;10:217-21.
73. Tokarz R, Williams SH, Sameroff S, Sanchez Leon M, Jain K, Lipkin WI. Virome analysis of *Amblyomma americanum*, *Dermacentor variabilis*, and *Ixodes scapularis* ticks reveals novel highly divergent vertebrate and invertebrate viruses*.* J Virol. 2014;88:11480-92.
74. Fryxell RT, Steelman CD, Szalanski AL, Kvamme KL, Billingsley PM, Williamson PC. Survey of Borreliae in ticks, canines, and white-tailed deer from Arkansas, U.S.A*.* Parasit Vectors. 2012; 5:doi: 10.1186/1756-3305-5-139.
75. Ullmann AJ, Gabitzsch ES, Schulze TL, Zeidner NS, Piesman J. Three multiplex assays for detection of *Borrelia burgdorferi* sensu lato and *Borrelia miyamotoi* sensu lato in field-collected *Ixodes* nymphs in North America*.* J Med Entomol. 2005;42:1057-62.
76. Walk ST, Xu G, Stull JW, Rich SM. Correlation between tick density and pathogen endemicity, New Hampshire*.* Emerg Infect Dis. 2009;15:585-7.
77. Williamson PC, Billingsley PM, Teltow GJ, Seals JP, Turnbough MA, Atkinson SF. *Borrelia*, *Ehrlichia*, and *Rickettsia* spp. in ticks removed from persons, Texas, USA*.* Emerg Infect Dis. 2010;16:441-6.
78. Yabsley MJ, Nims TN, Savage MY, Durden LA. Ticks and tick-borne pathogens and putative symbionts of black bears (*Ursus americanus floridanus*) from Georgia and Florida*.* J Parasitol. 2009;95:1125-8.
